# Hydrogen Spillover from Pt, Pd, and Au Evaluated with MnO_*x*_ Temperature-Programmed Reduction

**DOI:** 10.1021/jacsau.6c00822

**Published:** 2026-08-31

**Authors:** Mohammad Hamidizirasefi, Audrey M. Battiste, Angela Pathickal Abraham, Andersen P. Dimon, Bert D. Chandler

**Affiliations:** † Department of Chemical Engineering, 52469 The Pennsylvania State University, University Park, Pennsylvania 16802, United States; ‡ Department of Chemistry, The Pennsylvania State University, University Park, Pennsylvania 16802, United States

**Keywords:** hydrogen spillover, hydrogen mobility, Au catalysts, Pt catalysts, MnO_*x*_ reduction, temperature-programmed reduction, Kissinger analysis

## Abstract

Hydrogen spillover occurs when H_2_ adsorbs on an active metal and is then transferred to a reducible metal oxide. While spillover is involved in many catalytic reactions, fundamental studies of spillover have been limited by the paucity of experimental methods available to study it. Here, we develop an experimental temperature-programmed reduction (TPR) protocol to rapidly compare spillover from different metals (Pt, Pd, Au ) onto P25 titania, using MnO_*x*_ reduction probe reaction. X-ray photoelectron and UV–visible spectroscopies indicated reduction to MnO in the presence and absence of active metals, and quantitative H_2_ consumption measurements were consistent across all samples. Observed MnO_*x*_ reduction temperatures, which decrease by shifting as much as 150 °C, depend strongly on the metal loading and confirm H_2_ activation occurs at metal sites. Adding only 300 ppm Au caused the Kissinger apparent activation energy to decrease from 55 to <10 kJ/mol. Comparisons across metals and loadings indicate that MnO_*x*_ reduction is limited by H_2_ activation only at very low metal loadings and temperatures. Gold induces the largest shift in MnO_*x*_ reduction, indicating that it is the most competent spillover metal under these conditions. At moderately higher loadings (≥1 site/100 nm^2^), Pt and Au become equivalent, signaling that MnO_*x*_ reduction is no longer limited by H_2_ activation at higher metal loadings. Palladium is moderately slower, consistent with the generation of a PdH_*x*_ phase at low temperature. At higher active site loadings, MnO_*x*_ reduction depends primarily on the surface concentration of spillover H, providing a simple and useful approach for comparing catalysts.

## Graphical abstract

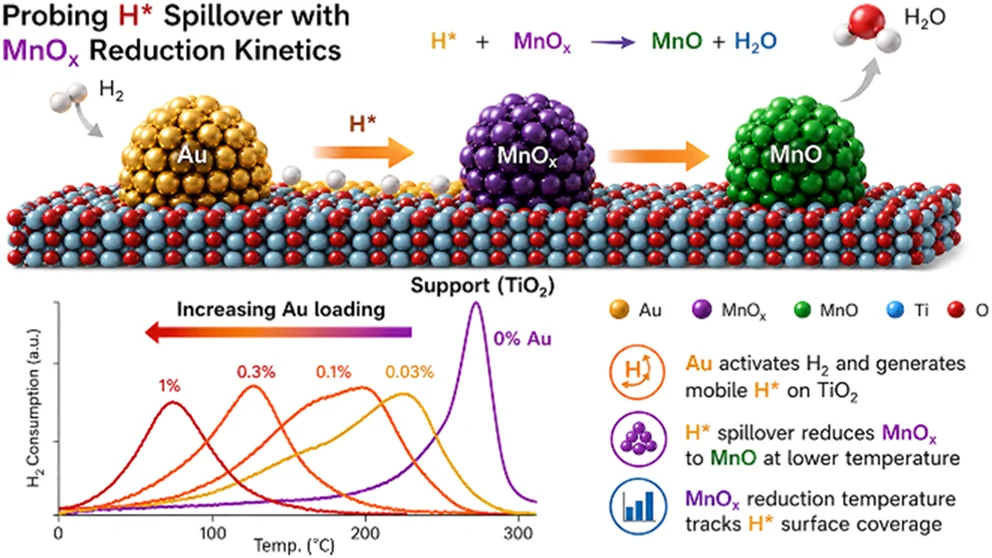

## Introduction

In 1964, Khoobiar first observed WO_3_ changes color when placed in contact with a Pt/Al_2_O_3_ catalyst and exposed to H_2_.^1^ He attributed the WO_3_ reduction to particle-to-particle migration of hydrogen atoms; Boudart et al. later coined the term “hydrogen spillover” to describe the phenomenon.^2,3^ Hydrogen spillover has been invoked in a multitude of reactions including selective hydrogenation of unsaturated hydrocarbons,^4,5^ ammonia production,^6^ CO/CO_2_ hydrogenation and methanation,^7,8^ methanol synthesis,^9^ water–gas shift reaction,^10,11^ Fischer–Tropsch synthesis,^12^ hydrodeoxygenation,^13^ and other dehydrogenations.^14–17^ Hydrogen spillover has also been examined in electrocatalytic hydrogen evolution and as a basis for hydrogen storage materials.^18,19^

While spillover was first reported in the context of support reduction, classic reviews by Prins, Conner, and Falconer highlight that several processes are involved in reducing the support.^20–22^ Interparticle support reduction requires the transport of a transient H species. These highly mobile H atom equivalents (H*), the active species in spillover, can rapidly traverse great distances across support surfaces.^2,3,20^ Karim et al. used clever electron beam lithography experiments to show H* can travel more than a micron across TiO_2_, whereas on Al_2_O_3_, H* migration was limited to a few nanometers.^23^ Shimakawa et al. measured spillover transport distances over ∼600 μm in a Pd/SrFeO_*x*_ system.^24^ Crossley et al. developed carbon nanotube array “hydrogen highways” to transport H* several microns between active Pd nanoparticles and CuO reduction targets.^25^

Similarly, Lee et al. examined how Pt influences H_2_ reactions on more-reducible CeO_2_ supports. STEM-EELS mapping showed H* populates regions far from the Pt-CeO_2_ interface. In this system, the H* electrons appear to be localized, resulting in reduced Ce^3+^ cations balanced by a surface proton, creating surface hydroxyls.^26^ The authors noted a clear difference between the localized spillover and support reduction. Even when the electron is localized on Ce^3+^, spillover is a reversible process, as both the Ce^3+^ and balancing hydroxyl disappear upon removing H_2_.

Support reduction, on the other hand, is an irreversible reaction between H* and the support. The loss of water removes the protons from the system, making this an irreversible process in the context of adsorption. Support reduction is more easily characterized rather than H*,^24,26–28^ leading to the two closely related phenomena often being conflated. Spillover is most prominent on reducible oxides, further adding to the confusion between the two processes.^23,27^

Transient, mobile, reactive species such as H* are difficult to quantify and characterize.^27,29,30^ In the case of spillover, the active species is best described as a loosely coupled proton and electron pair.^31–34^ The electron may be either localized on a specific cation (as on Pt/CeO_2_) or delocalized across multiple surface atoms.^26,31^ Mori and co-workers found that H* mobility depends strongly on support reducibility.^7,35^ Our previous work on titania suggests H* is highly mobile, even at near-ambient temperatures, and strongly dependent on the details of the support surface properties.^34,36,37^ Conversely, H* transport on Pt/CeO_2_ appears to be slower, requiring higher temperatures.

Several groups have used TPR to probe hydrogen spillover. Lee et al. combined H_2_-TPR with Kissinger analysis to quantify the apparent activation energy of Pt/CeO_2_ reduction. In that system, interfacial H* transfer from Pt to ceria was identified as a key kinetic step in spillover.^28^ Shun et al. systematically studied spillover by combining *in situ* X-ray absorption and H_2_-TPR of ZnO. They found that interparticle H^+^/e^–^ diffusion is facile on TiO_2_ and WO_3_ but saw limited H* mobility on MgO and CeO_2_.^38,39^ In a similar study, Beck et al. systematically examined H* spillover effectsfrom Pt supported on Fe_2_O_3_, CeO_2_, TiO_2_, ZrO_2_, and Al_2_O_3_ using H_2_-TPR. They found platinum significantly enhances CeO_2_ and Fe_2_O_3_ reduction but saw minimal effects on ZrO_2_ and Al_2_O_3_.^40^

Despite the importance of hydrogen spillover, direct experimental evidence capturing real-time mechanistic steps and kinetics remains limited. Herein, we develop an experimental protocol specifically designed to monitor the kinetic impact of hydrogen spillover on catalyst surfaces. By depositing a sacrificial metal oxide (MnO_*x*_) onto a catalyst containing a hydrogen activation metal (Au, Pd, Pt), we show that temperature-programmed reduction (TPR) quickly evaluates changes to the MnO_*x*_ reduction temperature. These changes can be attributed to the temperature-dependent H* surface coverage.

We further compare the relative activities of Pt, Pd, and Au in generating spillover. When the number of H_2_ activation sites per surface area is held constant, we find Au, which is not hindered by an oxide overlayer, is somewhat more effective at generating spillover than Pt or Pd. We also show that, above a certain threshold of H_2_ activation sites, both H_2_ activation and H* surface transport become fast relative to MnO_*x*_ reduction. Under these conditions, the MnO_*x*_ reduction temperature becomes a semiquantitative evaluation of the H* surface coverage. This foundational study provides a framework for the quantitative study of spillover using methods readily available to most catalysis researchers.

## Results and Discussion

### Spillover Test Development

#### MnO_*x*_ Deposition and Reduction

Mn­(III/IV) oxides (MnO_*x*_) are excellent H* reduction target candidates,^1^ as reduction to MnO is strongly exothermic (−74 to −127 kJ/molH_2_) (Figure S1).^41–43^ Briefly, we prepared O_*x*_Mn/TiO_2_ samples by slowly depositing Mn^2+^ onto freshly calcined TiO_2_. NH_4_OH was used to control pH during deposition, and samples were calcined in flowing air at 400 °C (see the Supporting Information (SI), S1.2).

The H_2_-temperature-programmed reduction (TPR) profiles (Figure 1A) of multiple O_*x*_Mn/TiO_2_ samples show two peaks centered at 240 and 310 °C. The relative peak areas (∼85% reduced below 270 °C) are similar to the fractions of anatase and rutile in this P25 (88% anatase). Both peak positions and normalized H_2_ uptakes (1.4 ± 0.1 H/Mn, Figure 1A, inset) are remarkably consistent across a broad range of Mn loadings. Control experiments varying the sample mass from 150 to 450 mg showed similar TPR profiles (Figure S4), indicating MnO_*x*_ reduction is not limited by external mass transfer.

**Figure 1:**
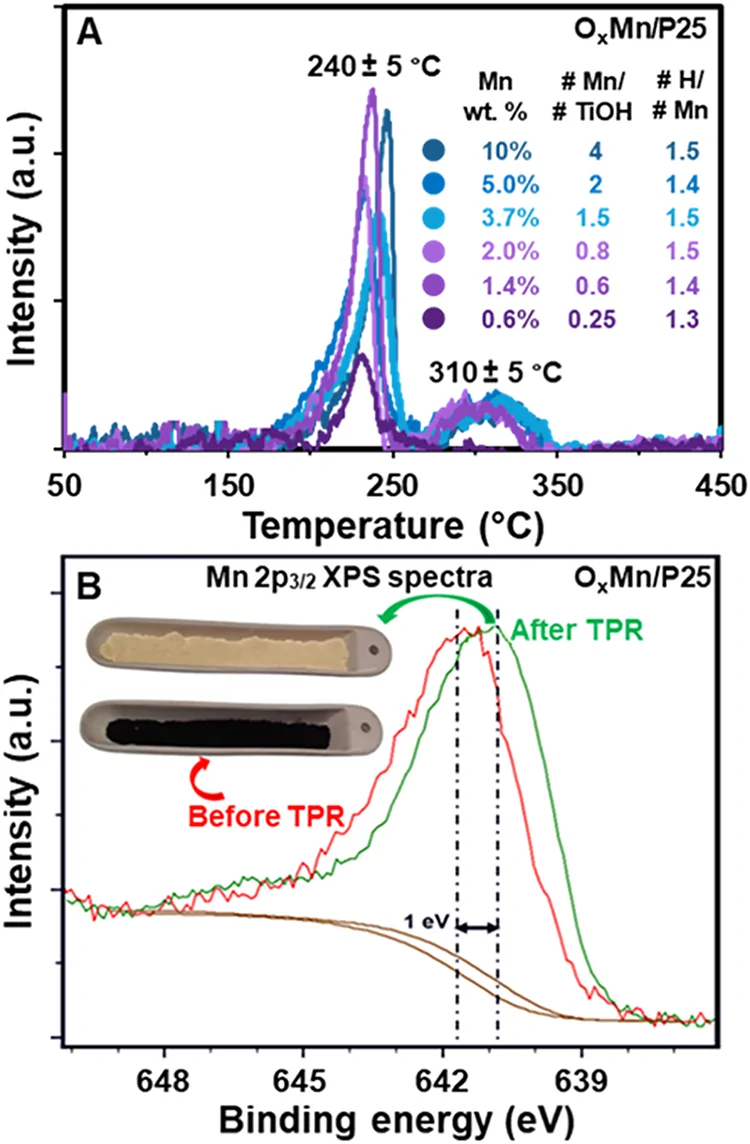
(A) H_2_-TPR profiles of O_*x*_Mn/P25 samples with varying Mn loadings. The inset table summarizes Mn loading (wt %), the corresponding equivalent Mn/TiOH ratio, and H/Mn uptake. Ti–OH density data were determined by thermal gravimetric analysis (TGA) and BET surface area analysis (Table S1, S1.2). Studies were performed under 5% H_2_/Ar with a 5 °C/min ramp rate. (B) Mn 2p_3/2_ X-ray photoelectron spectra (XPS) of O_*x*_Mn_1120_/P25 (5 wt % Mn) before (red) and after (green) TPR, showing a shift in binding energy (∼1 eV) upon reduction. The inset picture shows the sample color before (black) and after (yellow to green) TPR.

The X-ray photoelectron spectroscopy (XPS) spectrum for the freshly calcined O_*x*_Mn/P25 (Figures 1B, S13, and S14, Table S3) shows the Mn 2p_3/2_ peak at 641.8 eV with a shoulder at 642.5 eV, suggesting the surface is dominated by Mn­(III) and Mn­(IV) species.^44^ After TPR, the Mn 2p_3/2_ peak shifts to 640.8 eV. The sample color also changes dramatically, similar to the color change in bulk MnO_2_ after TPR. UV–visible spectroscopy shows the band gap values for fresh and reduced bulk MnO_2_ are about 1.5 and 3.5 eV, respectively (Figures S20 and S21), and are similar to the values reported by Gangwar et al.^45–49^ Thus, across a wide range of Mn loadings, the XPS, TPR, and UV–visible spectroscopy data indicate the preparation method consistently yields supported Mn­(III)/Mn­(IV) mixed oxide phases with average Mn oxidation state of approximately +3.5 (MnO_*x*_). Exposure to H_2_ above ∼200 °C readily reduced MnO_*x*_ to MnO.

We prepared a series of Au+O_*x*_Mn/TiO_2_ samples by sequentially depositing Au then MnO_*x*_, varying the Au and MnO_*x*_ loadings (see S1.2). Briefly, Au/P25 catalysts were first prepared using urea deposition-precipitation, as we have previously reported.^37,50–52^ Manganese­(II) was deposited on the Au/TiO_2_ catalyst and oxidized to the Mn­(III/IV) oxide as described above. Samples were then dried and recalcined (air, 300 °C, 30 min) in situ immediately prior to TPR studies. Our nomenclature system identifies each material based on the surface loading of both Au and MnO_*x*_, as described in Table 1.

**Table 1:** Materials and Nomenclature^b^,^c^

sample^a^	Mn wt %	$\frac{\mathrm{Mn}}{T{\mathrm{iOH}}^{b}}$	$\frac{\mathrm{Mn}}{100\;nm^{2}}$
O*_x_* Mn_140_/P25	0.62 ± 0.01	0.3	140
O*_x_* Mn_450_/P25	1.8 ± 0.1	0.8	450
O*_x_* Mn_1120_/P25	5.8 ± 0.1	2.0	1120
O*_x_* Mn_2230_/P_25_	9.6 ± 0.1	4.0	2230

sample	Au wt %	$\frac{{\mathrm{Au active sites}}^{c}}{100\;nm^{2}}$	$\frac{\mathrm{Mn}}{100\;nm^{2}}$
0.1Au + O*_x_* Mn_450_/P25	0.035 ± 0.001	0.06	450
0.2Au + O*_x_* Mn_450_/P25	0.090 ± 0.001	0.2	450
0.6Au + O*_x_* Mn_450_/P25	0.29 ± 0.01	0.6	450
2Au + O*_x_* Mn_450_/P25	0.95 ± 0.01	2.0	450

a The metal loading for each sample is expressed using three different units to ease comparisons.

b Surface hydroxyl (OH) densities were determined from TGA and BET surface area measurements.

c This is equal to the total metal loading.

Transmission electron microscopy (TEM) images, available in the SI (Figures S16–S17), were collected on the 1 and 0.3 wt % Au samples. MnO_*x*_ could not be resolved from the TiO_2_ support by Z-contrast STEM imaging. This is primarily due to the small difference in atomic number between Mn (*Z* = 25) and Ti (*Z* = 22). However, this also suggests an absence of large MnO_*x*_ agglomerates, which might be observable in TEM images. Figure 2 shows energy-dispersive X-ray spectroscopy (EDS) elemental mapping images of 2Au–O_*x*_Mn_450_/P25 (1 wt % Au, 2 wt % Mn). The Mn signal is weak due to the low Mn loading,but spatially uniform, further confirming MnO_*x*_ is highly dispersed across the TiO_2_ support.

**Figure 2:**
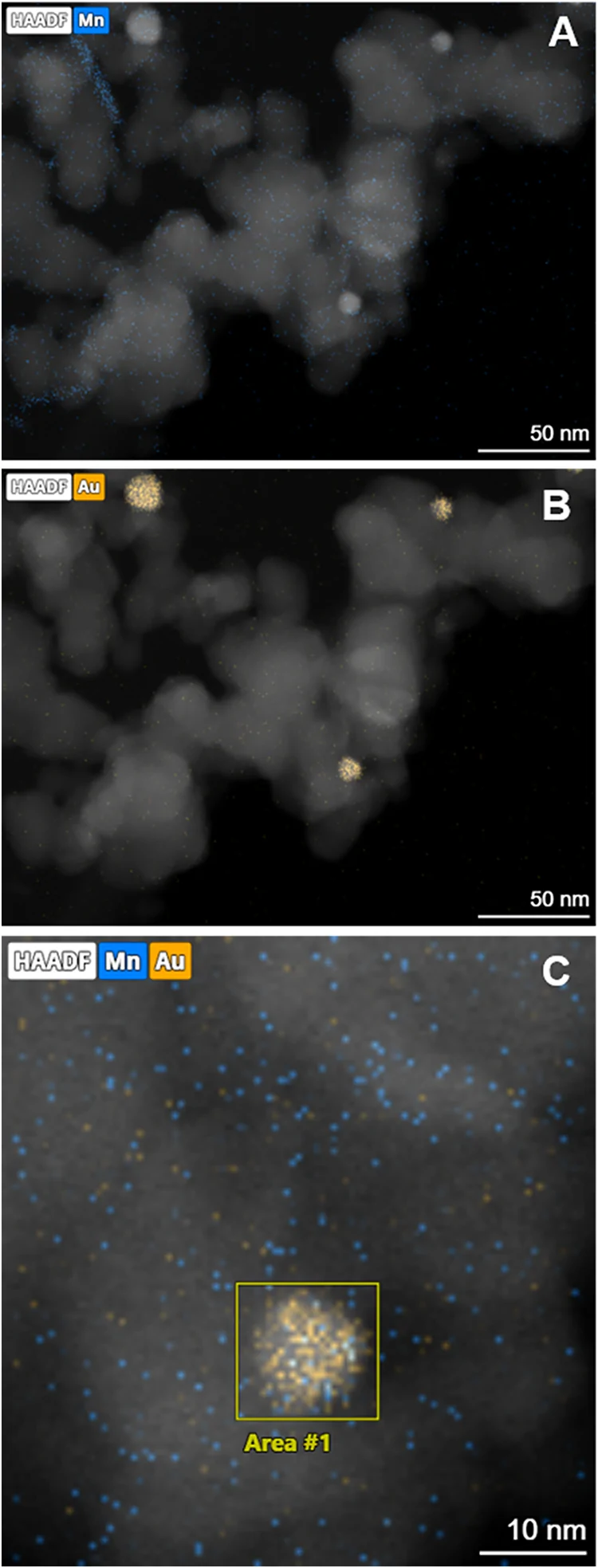
Representative EDS mapping images of the freshly calcined 2Au–O_*x*_Mn_450_/P25 (1 wt.% Au, 2 wt.% Mn) sample showing (A) MnO_*x*_ (blue), which is broadly dispersed across the TiO_2_ particles (gray), and (B) Au (yellow) localized as nanoparticles. (C) Magnification of the area near one of the Au particles, combining the Au and Mn EDS mapping. The EDS spectrum of the boxed area, available in the SI, shows only Au and Ti (no Mn).

We also performed X-ray diffraction on several samples to evaluate the MnO_*x*_ phase and crystallinity (Figure S15). At high Mn loadings (10 wt.% Mn; ∼4 Mn/surface TiOH), agglomerates broadly consistent with a pyrolusite phase of MnO_2_ are observed. XPS and TPR data for this sample (Figure S14, Table S3) are consistent with Figure 1, indicating a substoichiometric Mn (III/IV) oxide. Scherrer analysis indicates an average MnO_*x*_ crystallite size of approximately 7.5 nm. The 2 wt % MnO_*x*_ samples (∼0.8 Mn/TiOH) used in the spillover studies show no evidence of the corresponding pyrolusite phase. This indicates that any MnO_*x*_ domains are smaller than 5 nm or are completely absent in these materials; both interpretations are consistent with highly dispersed MnO_*x*_ on the titania.

The DS map shows the Au to be highly localized as cohesive nanoparticles (Figure 2B). The combined EDS map (Figure 2C) highlights the differences in metal distribution. Note the EDS spectrum of the boxed area in Figure 2C (Figure S16) shows only Au, indicating MnO_*x*_ is primarily deposited onto the titania. Z-contrast STEM images of the 1 and 0.3 wt % Au samples show the Au particles to be dispersed broadly across the support. Both samples had similar average Au particle sizes (5 nm ± 1.5) and distributions. Thus, the TEM/EDS data indicate the presence of both supported Au nanoparticles and widely dispersed MnO_*x*_ on the TiO_2_ support.

#### Au Loading Effects on MnO_*x*_ Reduction

Figure 3A shows MnO_*x*_ reduction occurs at much lower temperatures when Au is present. As little as 0.03 wt % Au lowers the first reduction peak ∼85 °C, and the MnO_*x*_ reduction peak consistently shifts to lower temperatures as Au loading increases. This shift in the peak maximum relative to the MnO_*x*_-only sample (0% Au–O_*x*_Mn/P25) is plotted in Figure 3B. On 1% Au samples, irreversible H_2_ uptake begins near room temperature, and the peak max occurs nearly 150 °C lower than the O_*x*_Mn/P25 samples (0% Au) (Figures 3A and S5). All shifts in the TPR peak maximum were essentially the same using 5 or 15 °C temperature ramps (Figure S5 A,B).

**Figure 3:**
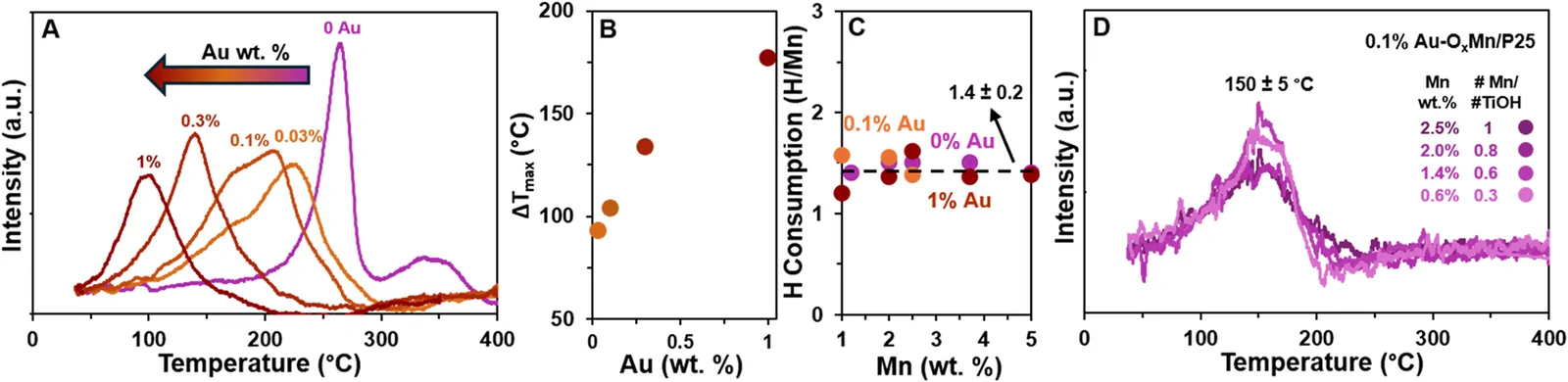
Impact of Au and Mn loading on MnO_*x*_ reduction kinetics. (A) H_2_-TPR profiles measured on Au + O_*x*_Mn_450_/P25 catalysts with varying Au loading (0, 0.03, 0.1, 0.3, 1 wt % Au) at a constant Mn surface density (∼450 Mn/100 nm^2^, 2 wt % Mn). (B) MnO_*x*_ reduction peak maximum shift (Δ*T*_max_) plotted vs Au loading (wt %). Studies were performed under 5% H_2_/Ar with 15 °C/min ramp rates. Data extracted from plot A. (C) H_2_ consumption (H/Mn) by O_*x*_Mn/P25 and Au + O_*x*_Mn/P25 catalysts plotted vs Mn loading. (D) Effect of Mn loading on MnO_*x*_ reduction kinetics in the presence of Au; H_2_-TPR profiles measured on 0.1% Au–O_*x*_Mn/P25 (0.2 Au-MSI active sites/100 nm^2^) catalysts with various MnO_*x*_ loadings (∼140, 280, 450, and 560 Mn atoms/100 nm^2^). Measurements in (C, D) were performed under 5% H_2_/Ar with a 5 °C/min ramp rate.

The H_2_ uptakes (1.4 ± 0.2 H/Mn, Figure 3C) remained consistent across all the Au and MnO_*x*_ loadings. Further experiments with constant Au loading (0.1% Au) and varying MnO_*x*_ loading (0.6–2.5 wt % Mn) showed essentially the same reduction peak max (∼150 ± 5 °C, Figure 3D). Total hydrogen consumption also remained consistent at ∼1.4 H/Mn across this series. A similar result was obtained using 1% Au and varying the MnO_*x*_ loading (Figure S3A). Thus, the initial Mn oxidation state and extent of reduction are consistent across all samples; the presence of Au only affects the MnO_*x*_ reduction kinetics. Note that the TPR experiment requires irreversible H_2_ consumption; therefore, the only observable in these experiments is the MnO_*x*_ reduction.

We evaluated the changes in reduction kinetics using H_2_-TPR Kissinger analyses.^28,53,54^ Figure 4A,B shows the representative ramp rate studies for samples without and with Au (Figures S6–S12); extracted apparent activation barriers (*E*_app_) are shown in Figure 4C. For simplicity, we limit our analysis to the dominant lower-temperature peak. The measured apparent reduction barrier for O_*x*_Mn_450_/P25 is 55 ± 5 kJ/mol, which is likely associated with the barrier for H_2_ activation on MnO_*x*_. This is slightly lower than values for bulk MnO_*x*_ reported by Sarkar^41^ (60–65 kJ/mol) and Bruijn^55^ (77–100 kJ/mol). Given the higher surface:bulk ratio of the dispersed MnO_*x*_ in our samples, a slightly lower apparent value is reasonable.

**Figure 4:**
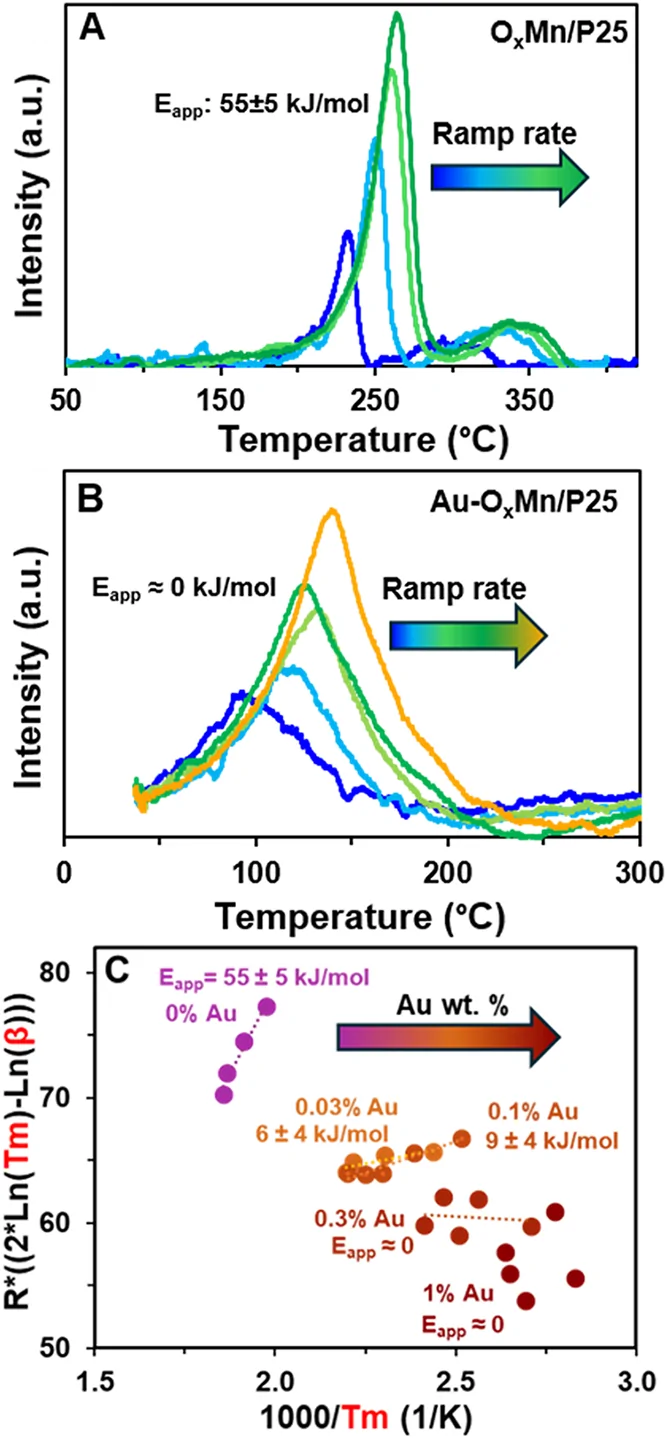
Kissinger analysis of H_2_-TPR reduction kinetics for O_*x*_Mn/P25 and Au–O_*x*_Mn/P25 catalysts. (A) H_2_-TPR profiles and corresponding peak maximum (*T*_m_) shifts for O_*x*_Mn_450_/P25 (0% Au) sample measured under 5% H_2_/Ar at different ramp rates (β): 5–15 °C/min. (B) H_2_-TPR profiles and peak max (*T*_m_) shift for 0.3% Au+O_*x*_Mn_450_/P25 (0.6 Au sites/100 nm^2^; ∼450 Mn atoms per 100 nm^2^) measured under 5% H_2_/Ar at various ramp rates (β): 5–15 °C/min. (C) Summary of apparent activation energy (*E*_app_) evaluated by Kissinger analysis of the low-temperature peak for O_*x*_Mn_450_/P25 and Au + O_*x*_Mn_450_/P25 catalysts with various Au loadings ∼0, 0.03, 0.1, 0.3, and 1 wt % Au and similar ∼450 Mn/100 nm^2^ (2 wt % Mn) (detailed in Table 1, S11).

Adding just 300 ppm Au (0.03 wt %, 0.1 MSI sites/100 nm^2^) decreases the *E*_app_ for MnO_*x*_ reduction to <10 kJ/mol; higher Au loadings yield a *E*_app_ ≈ 0. An apparent activation barrier near 0 is consistent with the kinetic parameters (H_2_ activation and H* transport) being relatively fast and MnO_*x*_ reduction being limited by the H* surface concentration. Note, care should be taken in interpreting Kissinger *E*_app_ values because they are associated with changes in the temperature of the maximum reduction rate (*T*_max_), not intrinsic rate constants.^28,56,57^ In this case, the Kissinger *E*_app_ values incorporate the kinetics of H_2_ activation, H* surface mobility, and MnO_*x*_ reduction, all of which have temperature dependences. Thus, while the differences in *E*_app_ values clearly show that Au dramatically improves the MnO_*x*_ reduction kinetics (Figures 4C and S12), they should be interpreted as highlighting the trend and may not necessarily correspond to a physically meaningful value.

These results are all consistent with MnO_*x*_ reduction occurring through a Au-catalyzed spillover mechanism, as depicted in Scheme 1. In this process, H_2_ is first activated at Au/TiO_2_ interface sites (Au-MSI), yielding 2 H*; the H* then diffuses across TiO_2_ and reacts with MnO_*x*_. The dependence on Au loading is particularly compelling evidence for this mechanism as the increased MnO_*x*_ reduction rates must be due to H_2_ activation by Au.

**Scheme 1:**
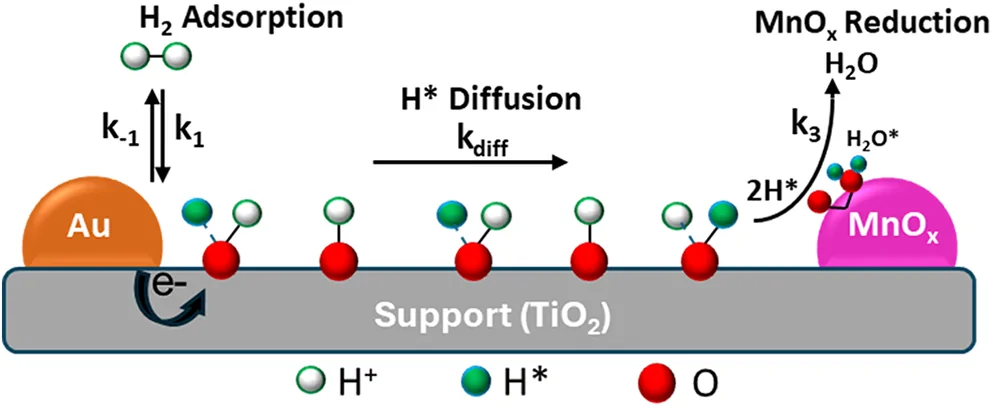
Illustration of the Spillover-Mediated MnO_*x*_ Reduction Mechanism^a^

a H_2_ is first activated at the Au/TiO_2_ interface, yielding H*, which diffuses across the TiO_2_ support. The subsequent reaction between H* and MnO_*x*_ yields MnO and H_2_O.

As Scheme 1 shows, the three reaction steps are governed by four underlying reaction parameters, each of which changes with temperature: (i) the rate constant for H_2_ activation and adsorption (H_2_ pressure is constant in the TPR experiment); (ii) H* surface mobility; (iii) the equilibrium H* coverage on the support; and (iv) the rate constant for MnO_*x*_ reduction by H*. Note that the last two steps combine to define the MnO_*x*_ reaction rate.^23,26,28,34,38^

We have previously shown the H_2_ activation rate constant at the Au/TiO_2_ interface is independent of particle size.^52^ However, increasing the Au loading increases the number of H_2_ activation sites, and therefore the H_2_ total activation rate at all temperatures.^52^ The equilibrium surface H* concentration on P25 also increases with temperature.^31,34,37^ With so many temperature-dependent factors, a formal kinetic evaluation of each step is not possible with temperature-programmed reduction; this is highlighted by the Kissinger analyses. In this context, TPR studies are perhaps more readily conceptualized aslight-off curves for spillover—the data do not lend themselves to full kinetic analysis, but nonetheless contain information on the key kinetic steps.

Five observations indicate H* diffusion across titania is fast relative to H_2_ activation. First, Au/P25 catalysts equilibrate H_2_ and H* in ∼10 min, with ∼80% of the adsorption occurring in the first ∼2–3 min.^37,52^ Second, previously published kinetics data show the H_2_ adsorption rate is constant before and after the MSI sites are saturated with H*. This is only possible if H* transport away from the interface is fast relative to H_2_ activation.^52^

Third, decreasing the TPR ramp rate to 5 °C/min has little to no impact on the onset of MnO_*x*_ reduction. This is most easily seen in Figure 4B, where the leading edge of the peak is essentially identical regardless of the ramp rate; this is true for all the Au loadings (Figures S7–S10). If the titania surface was not fully equilibrated with H* (or nearly fully equilibrated), the onset of MnO_*x*_ reduction should start at lower temperatures during the slower ramp rate experiments. Fourth, as noted above, the Kissinger *E*_app_ value for MnO_*x*_ reduction is approximately zero; this is consistent with a thermodynamically limited process rather than one limited by H* diffusion.

Finally, the MnO_*x*_ reduction temperature is independent of MnO_*x*_ loading at 0.1 wt % Au (Figure 3D). For these samples, the average Au-MnO_*x*_ distance, and thus the average H* diffusion distance, decreases as the MnO_*x*_ loading increases. The H_2_ activation kinetics, H* transport, and H* surface concentration change consistently with temperature across all samples. If H_2_ activation or transport were rate limiting under these conditions, the MnO_*x*_ reduction temperature should decrease with increased MnO_*x*_ loading. However, the MnO_*x*_ reduction reaction temperature is constant, beginning when the H* coverage reaches a critical concentration regardless of how many MnO_*x*_ sites are present.

This has important consequences for interpreting the TPR temperature shifts. In the context of a TPR experiment, the sample is constantly under H_2_, and the H* coverage changes are incremental with temperature. It is therefore reasonable to conclude the H* coverage is close to the equilibrium provided H_2_ activation is sufficiently fast to supply the equilibrium H* coverage.

#### H* Supply from Other Metals

We extended this protocol to Pt and Pd to assess its broader applicability and evaluate the relative kinetics of H_2_ activation, H* transport, and MnO_*x*_ reduction by H*. Sample details (metal loadings, elemental analyses) are available in Table S2. Hydrogen activation is widely considered to be much faster on Pt and Pd; however, two fundamental differences in the metals complicate direct comparisons between the systems. First, both Pt and Pd are at least partially oxidized from the pretreatment used to prepare the MnO_*x*_ reporter species.^58–63^ The TPR experiments necessarily incorporate reduction of the nascent surface oxide.

Second, all surface Pt and Pd atoms activate H_2_, while only Au/TiO_2_ interface sites are competent for H_2_ activation.^31,50,64–67^ To properly compare the metals and deal with the lower atomic weight of Pd, we used the TEM data to model the metal particles as cuboctahedra and estimate the number of active sites on each catalyst.^34^ Details of these calculations, including a plot of the number of H_2_ activation sites with the measured weight loading (Figure S19), are available in the SI (S3.2). Most importantly, especially for Pt and Pd, the changes in metal loading, which cover more than an order of magnitude, have a greater impact on the number of active sites than even moderate differences in the metal particle size.

#### Active Site Comparisons

Figure 5A,B shows TPR measurements on supported Pt and Pd catalysts, respectively; the impact of active site density on Δ*T*_max_ is in Figure 5C. All the metals show the same general trend of Δ*T*_max_ increasing with metal loading. Several trends are of note. First, Au has the largest overall impact on the MnO_*x*_ reduction temperature. Even at low active site densities, adding Au decreases the MnO_*x*_ reduction temperature by nearly 100 °C; Pt and Pd induce shifts of <30 °C. This observation that Au is the most competent metal for fostering spillover under these conditions (low temperature, low active site density) is somewhat surprising.

**Figure 5:**
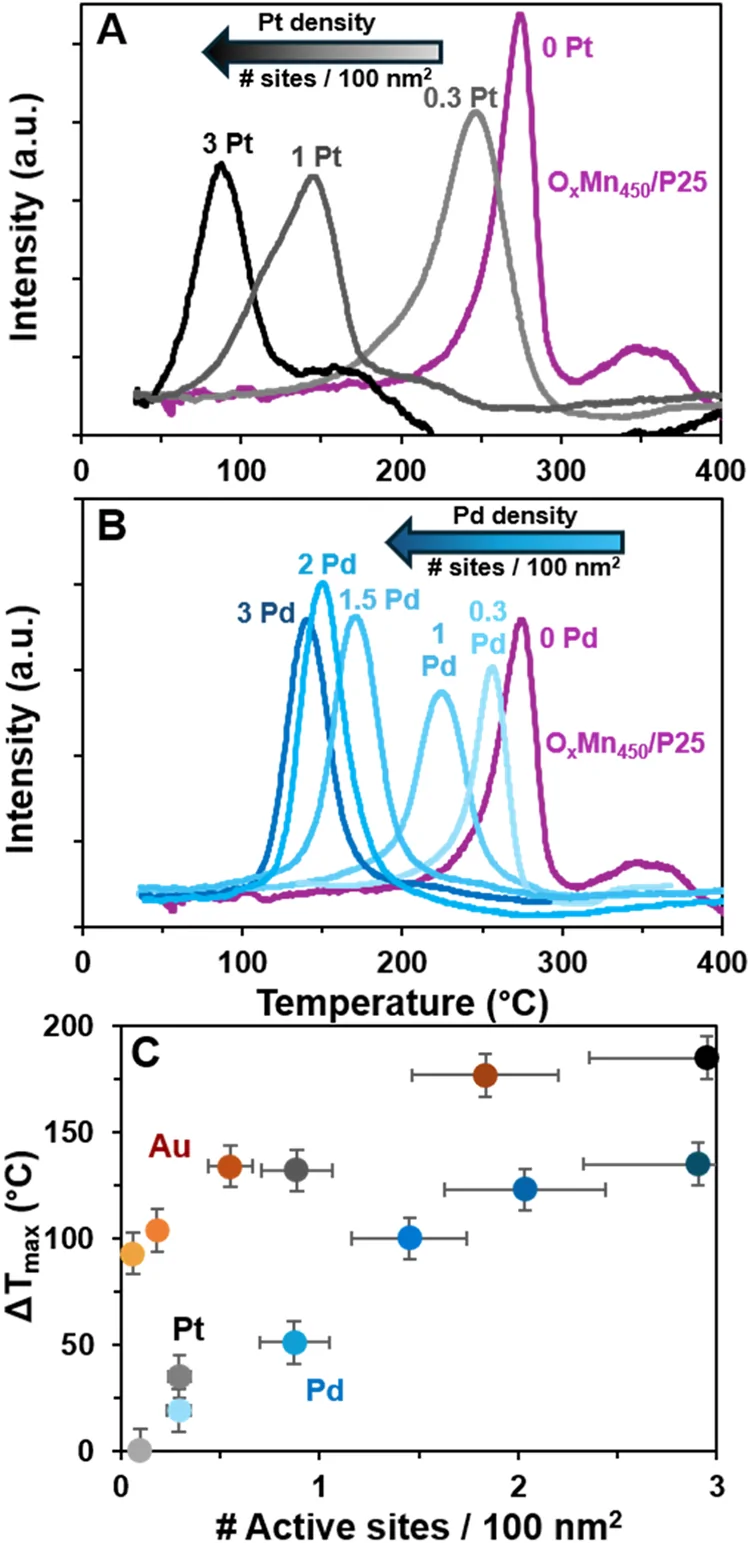
Impact of H_2_ activation metal and active site surface density on MnO_*x*_ reduction kinetics. H_2_-TPR profiles measured on (A) Pt + O_*x*_Mn_450_/P25 and (B) Pd + O_*x*_Mn_450_/P25 catalysts with varying metal surface densities (0–3 active sites/100 nm^2^ cat.) at a constant Mn surface density (∼450 Mn/100 nm^2^ density, 2 wt % Mn). (C) Shift in MnO_*x*_ reduction peak temperature (Δ*T*_max_) as a function of H_2_ activation site density for Au, Pt, and Pd. Studies were performed under 5% H_2_/Ar with a 15 °C/min ramp rate.

For Pt, the Δ*T*_max_ values increase roughly linearly with metal loading, then plateau. Above ∼0.7 sites/100 nm^2^, Pt and Au have similar Δ*T*_max_ values, decreasing the MnO_*x*_ reduction temperature by ∼180 °C. Pd shows the same general trends, but is consistently less effective at lowering the MnO_*x*_ reduction peak max and plateaus at a Δ*T*_max_ value of ∼140 °C.

These results shed light on how to utilize MnO_*x*_ reduction to evaluate spillover. We first examine the intrinsic differences between metals and the unexpected observation that Au is the most effective metal at lowering the MnO_*x*_ reduction temperature. The origins of this effect are unclear, but two explanations are consistent with the observations.

First, Au activates H_2_ directly at the MSI, yielding two H* atoms on support hydroxyls.^51^ Pt and Pd, on the other hand, adsorb H_2_ on metal sites, requiring a transfer of H* from the metal to the support. The presence of a kinetic barrier to this transfer, especially at low temperatures, is consistent with the Pt lower apparent activity. Additionally, oxygen adsorption on reduced Au surfaces is endothermic,^52,68^ leaving the interface free to activate H_2_ at low temperatures. After the oxidation step required in MnO_*x*_ deposition, the Pt particles have a nascent oxide surface layer^69^ that must be removed—removal of this oxide layer might account for the smaller Δ*T*_max_ values.

The PdO_*x*_/P25 TPR data in Figure 6 show a large H_2_ desorption peak at ∼75 °C with a H/Pd ratio of 0.7. Cryogenic TPR experiments by Ryu et al.^70^ and Neumann et al.^71^ showed that PdO_*x*_ is reduced at much lower temperatures than PtO_*x*_ and assigned the H_2_ evolution to the decomposition of a PdH_*x*_ compound.^70–73^ Our data are consistent with these observations, which provide an explanation for why the Pd Δ*T*_max_ values consistently lag behind Pt and Au. It appears the PdH_*x*_ compounds cannot catalytically activate H_2_ beyond their stoichiometric values, and the PdH_*x*_ must decompose to catalytically active metallic Pd before H* can be transferred to the support. This interesting underlying chemistry makes Pd somewhat less useful for examining the role of spillover at lower temperatures. However, the broad conclusion that the H* surface concentration on titania is likely the same for all three metals seems reasonable with this minor caveat regarding the decomposition of PdH_*x*_.

**Figure 6:**
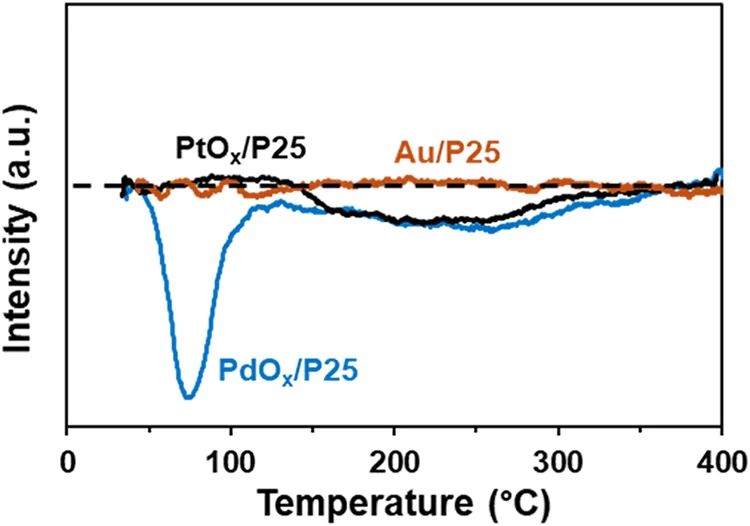
H_2_-TPR profiles measured on peroxidized PtO_*x*_/P25 (5 wt % Pt), PdO_*x*_/P25 (5 wt % Pd), and Au/P25 (1 wt %). Studies were performed under 5% H_2_/Ar with 15 °C/min ramp rates.

Finally, while these differences become apparent under the experimental conditions required for these measurements, they appear to be primarily observable because of the low temperatures and metal loadings. At higher temperatures, where most catalytic reactions occur, we expect the differences between metals to become negligible. This is consistent with spillover ultimately being a property of the support rather than that of the metal.

#### Relative Rates of H_2_ Activation, H* Transport, and MnO_*x*_ Reduction

The Pt and Au data provide an opportunity to evaluate the relative roles of H_2_ activation kinetics, H* transport, and MnO_*x*_ reduction kinetics. The activation barrier for H_2_ adsorption at Au/TiO_2_ interfaces is low (∼20 kJ/mol), and the intrinsic H_2_ activation kinetics at the Au/TiO_2_ interface are independent of Au particle size.^52,68^ However, at infinitely low metal coverage, H_2_ activation must become rate limiting. At sufficiently high loadings, there will be enough H_2_ activation sites to rapidly equilibrate the surface. The salient question then becomes: what is the minimum active site loading to ensure the surface is nearly equilibrated with H*?

Similarly, the H* diffusion constants, the equilibrium H* coverage at any given temperature, and the intrinsic MnO_*x*_ reduction kinetics are properties of the support, and therefore independent of the metal or loading. This leaves only two related parameters that can reasonably change as metal loading increases: (i) the number of H_2_ activation sites, which increases the global H_2_ activation rate, and (ii) the surface concentration of H* at a given temperature. The H_2_-TPR experiment is a dynamic system as all the kinetic parameters and the equilibrium H* surface concentration increase with temperature; however, general conclusions can be made. At low Au loadings, the small number of MSI sites are unable to supply enough H* to approach the equilibrium H* coverage. Under these conditions, MnO_*x*_ reduction is limited by the H_2_ activation kinetics; a nonzero *E*_app_ value is observed in the Kissinger analysis, and a smaller Δ*T*_max_ (higher MnO_*x*_ reduction temperature) is observed.

At higher Au loadings, Δ*T*_max_ becomes less sensitive to Au loading, indicating H_2_ activation is no longer rate limiting. This is supported by the convergence of the Au data with the Pt data at ∼1 site/100 nm^2^. Presumably, the rate constant for H_2_ activation on Pt is faster than at the Au/TiO_2_ MSI. The point at which the Δ*T*_max_ values converge can therefore be evaluated as the minimum point, where H_2_ activation is no longer rate determining. This is further supported by the fact that Pt and Au show nearly the same Δ*T*_max_ values at 3× higher loadings. Because the equilibrium H* surface concentration also increases with temperature, it is difficult to evaluate the minimum Au loading where H_2_ activation limits the H* supply. While the true value is almost certainly somewhat lower than 1 site/100 nm^2^, under these conditions, it is safe to conclude H_2_ activation is fast.

Above this threshold, MnO_*x*_ reduction appears to be governed by the H* surface concentration and the rate constant for the reaction between MnO_*x*_ and H*. At high metal loadings, where H_2_ activation and H* transport across the support is fast, the surface is equilibrated (or nearly equilibrated) with H*. Hydrogen adsorption experiments performed via infrared spectroscopy indicate Au/P25 surfaces adsorb ∼80% of the total H_2_ uptake in ∼3 min and are essentially fully equilibrated in ∼5 min.^37,52^ In the context of the TPR experiment, where the H* surface concentration under a constant H_2_ flow increases incrementally as the temperature increases,^37,74^ it is therefore reasonable to conclude the surface is largely equilibrated once the H_2_ activation kinetics are sufficiently fast (above 1 site/100 nm^2^).

Combining all these observations, under conditions where H_2_ activation and H* transport across the support are fast, the MnO_*x*_ reduction kinetics appear to be controlled by, and therefore a semiquantitative measure of, the H* surface concentration. Moreover, this analysis establishes as relatively simple TPR method for evaluating and comparing spillover across metals and supports. Future measurements should allow us to establish clear measures of an individual support’s relative reactivity toward H*, and provide concrete guidance for when H*-based mechanisms should be considered in a given catalytic reaction.

## Conclusions

We developed a semiquantitative measure for hydrogen spillover on titania utilizing a metal (Pt, Pd, Au) to activate H_2_ and surface MnO_*x*_ as a reporter molecule. TPR studies showed the MnO_*x*_ reduction temperature depends strongly on the metal loading, and Kissinger studies showed dramatic changes in *E*_app_ for Au samples. These results confirm H_2_ is activated at metal sites, transported across the titania as H*, and facilitates MnO_*x*_ reduction.

When evaluated using similar numbers of H_2_ activation sites, Au was more competent at fostering spillover than Pt or Pd—particularly at low active site densities. At higher active site densities, Pt and Au become equivalent while Pd lags in apparent activity. The lower Pd competence appears to be due to the generation of a Pd hydride phase at low temperature, which must decompose before spillover can occur.

The MnO_*x*_ reduction process involves three steps: H_2_ activation, H* transport, and MnO_*x*_ reduction. At high loadings, where Pt and Au show nearly identical behavior on a per active site basis, our study indicates H_2_ activation and H* transport are likely fast. Under these circumstances, MnO_*x*_ reduction appears to be governed primarily by the H* surface concentration, making the TPR tests rapid semiquantitative measures of the support’s ability to stabilize spillover.

## Materials and Methods

### Chemicals

UHP-grade argon, oxygen, and 5% H_2_/Ar gases were supplied by Pennsylvania State University gas supplier (Linde Gas). TiO_2_–P_25_ nanopowder (49 m^2^/g surface area, 0.47 cm^3^/g pore volume) was supplied by Evonik. HAuCl_4_·3H_2_O (99.7%), (NH_4_)_2_PtCl_4_ (99%, Mw: 372.97 g/mol, CAS No.: 13,820–41–1, solid), and MnSO_4_·H_2_O (>99%, CAS No.: 10,034–96–5) were purchased from Sigma-Aldrich. Palladium­(II) chloride (99.9% Pd, CAS No. 7647–10–1) was supplied by STREM Chemicals. Concentrated NH_4_OH (29.3 wt % NH_3_, 14.5 M) was purchased from Fisher Scientific. Urea (99.5%) was purchased from Acros Organics.

### Catalyst Synthesis

#### Au/TiO_2_–P_25_

The 1% Au/TiO_2_ catalyst was synthesized by the urea deposition-precipitation method reported in our previous works.^36,37,74^ The support was calcined at 400 °C for 3 h beforehand. For a 2 g sample, HAuCl_4_ (40 mg) and urea (200 mg) were dissolved in 35 mL of deionized (DI) H_2_O in a round-bottom flask. The yellow solution was heated while stirring until the temperature reached 60 °C (10–15 min). Then, 2 g of TiO_2_ was added, and the mixture was stirred at 80 °C for 4 h. After 4 h, the stirring was stopped, and the mixture was cooled to room temperature. The solid was isolated by centrifugation at 7000 rpm and washed with 5 × 30 mL H_2_O, 40 mL 0.1 M NH_4_OH, and then further washed with 5 × 30 mL DI H_2_O until the filtrate reached pH ∼ 7. Upon the final wash, the filtrate was tested with AgNO_3_ to confirm the absence of chloride ions. The solid was then dried at 90 °C in an oven overnight and subsequently reduced at 275 °C under flowing 5% H_2_/N_2_ for 4 h. The catalyst was cooled to room temperature and stored at 4 °C in air.

#### O_*x*_Mn/Supports, Au–O_*x*_Mn/Supports

These samples were synthesized using a combination of strong electrostatic adsorption (SEA) and deposition-precipitation with NH_4_OH, developed for the first time by our group. Before synthesis, the corresponding primary samples/supports (TiO_2_, Au/TiO_2_) were calcined at 400 °C for 3 h to remove any volatiles and clean the surface for Mn addition. The point zero charge (PZC) of the supports was determined, and the synthesis pH was set between the PZC and 1 unit above the PZC to ensure slow deposition and negative surface charge to adsorb Mn^2+^. To prepare a 1 g sample, the supports or Au/Support samples were added to 30 mL of deionized (DI) water and mixed for 30 min in a beaker. Then, an adequate amount of NH_4_OH was added to adjust the pH to the range of 7–8 for TiO_2_. Meanwhile, a defined amount of MnSO_4_·H_2_O salt was dissolved in water to make the salt precursor and stirred for 30 min. The concentration of the Mn salt precursor was adjusted to about 0.015 M Mn for all samples, which results in a very low surface loading (400–600 m^2^/L), ensuring good dispersion and easier pH control, as recommended in previous studies on deposition-precipitation methods. SEA preparation method usually yields a much smaller particle size with much smaller standard deviations.^75^

The salt solution was then added very slowly and dropwise to the support suspension under vigorous stirring. The rate of adding the Mn solution was maintained at 10 droplets/min to ensure good dispersion and prevent formation of large agglomerates in the solution or on the support surface. After the Mn solution was fully added to the support suspension, the pH was adjusted to the upper limit of the target range to make sure all Mn precursors were added, and the suspension was stirred for 1 more hour. Then, hydrogen peroxide H_2_O_2_ was added in a 3-fold molar excess to promote the oxidation of Mn­(OH)_2_ to MnO_*x*_, followed by continued stirring for an additional 2 h. The solid was separated using centrifugation and washed repeatedly with water (five times) until minimal ionic residue remained and the pH of the wash solution reached 7. The resulting precipitate was then dried overnight and calcined at 400 °C for 3 h.

#### Pt/TiO_2_ and Pt-MnO_*x*_/TiO_2_

The 0.07% Pt/TiO_2_–P25 (3 sites/100 nm^2^ support) catalyst was synthesized using the conventional wet impregnation method. One gram of support was suspended in 30 mL of DI-H_2_O under vigorous stirring at 400 rpm. Standard precursor solutions (0.02 and 0.002 mg salt/ml DI H_2_O) were used to make all low-loading Pt and Pd samples. A specific amount of Pt salt (NH_4_)_2_PtCl_4_, a volume equivalent to 1.4 mg salt for 0.07 wt % Pt, was added to 10 mL of DI-H_2_O. The temperature of the support suspension was raised to 70 °C, and the Pt precursor solution was gradually added at a rate of 1 mL/min. The mixture was stirred for 1 h at 70 °C, then heated to 90 °C under continuous stirring for 12 h to allow complete drying and water evaporation. The dried powder was then ground and reduced at 400 °C in 5% H_2_/N_2_ for 3 h to remove chlorine and other residual ions, followed by calcination in air at 400 °C for 3 h to prepare the sample for TPR or for MnO_*x*_ deposition on Pt/TiO_2_ (Pt-MnO_*x*_/TiO_2_). After reduction, the catalyst color changed to a grayish hue, and after calcination, it returned to a white color.

The 0.07% Pt- 2% Mn/TiO_2_ catalysts were synthesized similarly to the MnO_*x*_/TiO_2_ or Au-MnO_*x*_/TiO_2_ samples.

### H_2_-Temperature-Programmed Reduction (H_2_-TPR)

The H_2_-TPR measurements were performed using a Micromeritics AutoChem 2910 instrument. First, a specific amount of finely powdered sample was loaded into a U-shaped quartz tube, positioned on top of quartz wool. Prior to the H_2_-TPR measurement, the sample was reoxidized *in situ* using UHP O_2_ at a flow rate of 10 mL/min. This step aims to remove any adsorbed carbonates and clean the sample by heating it from room temperature to 300 °C at a ramp rate of 10 °C/min for 30 min. The sample was then cooled to ambient temperature in UHP O_2_ gas. Once at room temperature, the sample was purged with UHP Ar at a flow rate of 30 mL/min for 30 min to eliminate any residual or physisorbed oxygen before introducing hydrogen, thus preventing the formation of surface water due to H_2_-O_2_ interaction. Subsequently, a 5 vol % H_2_/Ar mixture was introduced at a flow rate of 50 mL/min as the carrier gas. After allowing the baseline to stabilize for 20 min, the sample was heated from ambient temperature to 500 °C with desired ramp rates (5–15 °C/min). A cold trap was placed in the gas line between the sample tube and the thermal conductivity detector (TCD) to capture any water released during the reduction process. The TCD intensity was calibrated using a standard CuO sample to calculate peak area or H_2_ consumption.

Further details of the materials and methods used are available in the SI.

## Supplementary Material



## Notes

Materials and methods, catalyst synthesis, H_2_-TPR traces, calculations, Kissinger analysis, and materials characterization data (material composition (XRF), XPS, XRD, bandgap analysis (UV–vis), NP size analysis, HAADF-STEM images, and EDS mapping) (PDF)
