# Supplementary material for: Hydrogen Spillover from Pt, Pd, and Au Evaluated with MnO x Temperature-Programmed Reduction
Source: JACS Au. 2026 Aug 31;6(9):5179–89. doi: 10.1021/jacsau.6c00822 (PMC13625579; doi:10.1021/jacsau.6c00822)
Supplement: Supplementary file 1 [file au6c00822_si_001.pdf]

Hydrogen spillover from Pt, Pd, and Au evaluated with MnO_x temperature programmed reduction

Mohammad Hamidizirasefi[1], Audrey M. Battiste[2], Angela Pathickal Abraham[2], Andersen P. Dimon[2], and Bert D. Chandler* [1,2]

[1]Department of Chemical Engineering, The Pennsylvania State University, University Park, Pennsylvania, 16802, United States.
[2]Department of Chemistry, The Pennsylvania State University, University Park, Pennsylvania, 16802, United States.

*To whom correspondence should be addressed: bert.chandler@psu.edu; phone (814) 863-6229

Contents:

1. Materials and methods ... 3
   - 1.1. Chemicals ... 3
   - 1.2. Synthesis ... 3
   - 1.3. Thermodynamics calculation for metal-oxides reduction in H_2 ... 4
   - 1.4. H_2-Temperature-Programmed Reduction (H_2-TPR) ... 5
     - A. H_2-TPR analysis of MnO_x loading effect in presence of 1% Au/P25. ... 6
     - B. H_2-TPR analysis of MnO_x/P25 sample mass effect on MnO_x reduction peak max. 6
     - C. H_2-TPR analysis of Au loading effects on MnO_x reduction kinetics. ... 7
2. Kissinger analysis of MnO_x reduction E_{app} using H_2-TPR ... 8
   - 2.1. H_2-TPR at various ramp rates ... 8
   - 2.2. Peak fitting ... 9
3. Characterization ... 11
   - 3.1. X-ray fluorescence spectroscopy (XRF) ... 11
   - 3.2. Catalyst Surface Characterization ... 11
   - 3.2.A. X-ray photoelectron spectroscopy (XPS) ... 11
   - 3.2.B X-ray diffraction analysis (XRD) ... 13
   - 3.2.C. HAADF-STEM and EDS Element Mapping ... 15
   - 3.2.D. Au and Pt Active Site Analysis ... 21
   - 3.3. UV-Vis spectroscopy ... 22
4. References ... 25

1. Materials and methods

1.1. Chemicals

UHP grade Argon, Oxygen, and 5% H_2/Ar gases were supplied by Linde gas (Pennsylvania State University gas supplier). TiO_2-P25 nano-powder (49 m^2/g surface area, 0.47 cm^3/g pore volume) was supplied by Evonik. $HAuCl_4.3H_2O$ (99.7%), $(NH_4)_2PtCl_4$ (99%, Mw: 372.97 g/mol Cas#: 13820-41-1 solid), and $MnSO_4.H_2O$ (>99%, Cas#: 10034-96-5) was purchased from Sigma-Aldrich. Palladium (II) chloride (99.9% Pd, Cas#: 7647-10-1) supplied from STREM chemicals. Concentrated NH_4OH (29.3%wt NH_3, 14.5M) was purchased from Fisher Scientific. Urea (99.5%) was purchased from Acros Organics.

1.2. Synthesis

Synthesis of Au/TiO_2-P25

The 1% Au/TiO_2 catalyst was synthesized by the urea deposition-precipitation method reported in our previous work.[1, 2] The support was calcined at 400 °C for 3 hours beforehand. For a 2-gram sample, $HAuCl_4$ (40 mg) and urea (200 mg) were dissolved in 35 mL of deionized (DI) H_2O in a round bottom flask. The yellow solution was heated while stirring until the temperature reached 60 °C (10-15 minutes). Then, 2 grams of TiO_2 were added, and the mixture was stirred at 80 °C for 4 hours. After 4 hours, the stirring was stopped, and the mixture cooled to room temperature. The solid was isolated by centrifugation at 7000 rpm, washed with 5 × 30 mL H_2O, 40 mL 0.1 M NH_4OH, and then was further washed with 5 × 30 mL DI H_2O until the filtrate reached pH ~7.[3] Upon the final wash, the filtrate was tested with $AgNO_3$ to confirm absence of chloride ions. The solid was then dried at 90 °C in an oven overnight and subsequently reduced at 275 °C under flowing 5% H_2/N_2 for 4 hours. The catalyst was cooled to room temperature and stored at 4 °C under air.

Synthesis of O_xMn/ TiO_2, Au-O_xMn/ TiO_2

These samples were synthesized using a combination of Strong Electrostatic Adsorption (SEA) and deposition-precipitation with NH_4OH. Before synthesis, the underlying sample/support (TiO_2, Au/TiO_2) was calcined at 400 °C for 3 hours to remove any volatiles and clean the surface for Mn addition. To prepare a 1-gram sample, the TiO_2 support or Au/ TiO_2 samples were added to 30 ml of deionized (DI) water and mixed for 30 minutes in a beaker under vigorous stirring. The pH was adjusted to 7-8 with 0.2 M NH_4OH.

Manganese addition was through strong electrostatic adsorption (SEA), which has previously been shown to yield high dispersions.[4] In a separate beaker, the defined amount of $MnSO_4.H_2O$ salt was dissolved in water and stirred for 30 minutes. The concentration of the Mn salt precursor was adjusted to 0.015 M Mn for all samples; Mn loading was adjusted by changing the volume of freshly prepared Mn solution added to the Au/TiO_2 catalyst. The salt solution was then added dropwise (~ 10 droplets/min) to the support suspension under vigorous stirring.

After the Mn solution was fully added to the support suspension, the pH was adjusted to the upper limit of the target range, and the suspension was stirred for 1 more hour. Hydrogen peroxide was added in a fivefold molar equivalent relative to Mn excess to promote the oxidation of $Mn(OH)_2$ to MnO_x, followed by continued stirring for an additional 2 hours. The solid was separated using centrifugation and washed repeatedly with water (five times) until SO_4^{2-} and NH^{4+}

ions are removed confirmed by UV-Vis spectroscopy, and the pH of the wash solution reached 7. The resulting precipitate was then dried overnight and calcined in air flow at 400 °C for 3 hours.[5]

Synthesis of Pt(Pd)/TiO_2 and Pt(Pd)-MnO_x/TiO_2

The 0.07% Pt/TiO_2-P25 (3 sites / 100 nm^2 support) catalyst was synthesized using the conventional wet impregnation method. 1 gram of support suspended in 30 ml of DI-H_2O under vigorous stirring rate at 400 RPM. Standard precursor solutions (0.02 and 0.002 mg salt/mL DI H_2O) were used to make all low loadings of Pt and Pd samples. A specific amount of Pt salt $(NH_4)_2PtCl_4$, a volume equivalent to 1.4 mg salt for 0.07 wt.% Pt was added into 10 ml DI-H_2O. To make Pd precursor, a few droplets of 0.5M HCL solution were added slowly and dropwise to dissolve the $PdCl_2$ salt and the precursor temperature was kept at 50 °C . The temperature of the support suspension was raised to 70 °C, and the Pt precursor solution gradually added at a rate of 1 mL/min. The mixture was stirred for 1 hour at 70 °C, then heated to 90 °C under continuous stirring for 12 hours to allow complete drying and water evaporation. The dried powder was then grounded and reduced at 400 °C in 5% H_2/N_2 for 3 hours to remove chlorine and other residual ions, followed by calcination in air at 400 °C for 3 hours to prepare the sample for TPR or for MnO_x deposition on Pt/TiO_2 (Pt-MnO_x/TiO_2). After reduction, the catalyst color changed to a grayish hue, and after calcination, it returned to a white color.

The 0.07% Pt- 2% Mn/TiO_2 catalysts were synthesized similarly to the MnO_x/TiO_2 or Au-MnO_x/TiO_2 samples. The Mn precursor was deposited onto Pt/TiO_2-Rut using the SEA+ NH_4OH deposition-precipitation method.

Unit conversion for TiO_2 (TiOH) surface Mn coverage to Mn loading:

$$\#\,\text{Mn} = \frac{\text{mass of Mn(wt \% loading} \times \text{catalyst mass)} \times \text{Avogadro number}}{\text{MW(Mn)}} \quad \text{(S1)}$$

$$\#\,\text{Ti} - \text{OH} = \text{mass of the support} \times \text{surface area} \times \text{OH density.} \quad \text{(S2)}$$

Table S1: Support's surface area and OH density

Support	Surface area(m^2/g)	OH density (#/m^2) (TGA)[a]
P25	50	5.5
Rutile	34	5.3
Anatase	13	8

[a] Data were collected by TGA in N_2.

1.3. Thermodynamics calculation for metal-oxides reduction in H_2

ΔG values in **Figure S1** were calculated using Eq. (1) by considering the H_2 concentration in H_2-TPR analysis and assuming very low H_2O content during the reduction. The standard

$\Delta G_r = \Delta G° + RT \ln Q = \Delta H° - T\,\Delta S° + RT \ln (P_{H_2O}/P_{H_2}$

thermodynamic values for formation enthalpy and entropy were extracted from NIST data base and the data provided by OpenStax Rice University.[6]

(S3)

Assumption:

$P_{H2O}/P_{H2} = 0.001/0.05$

MnO_x reduction is thermodynamically favorable even at room temperature [7], [8], [9].

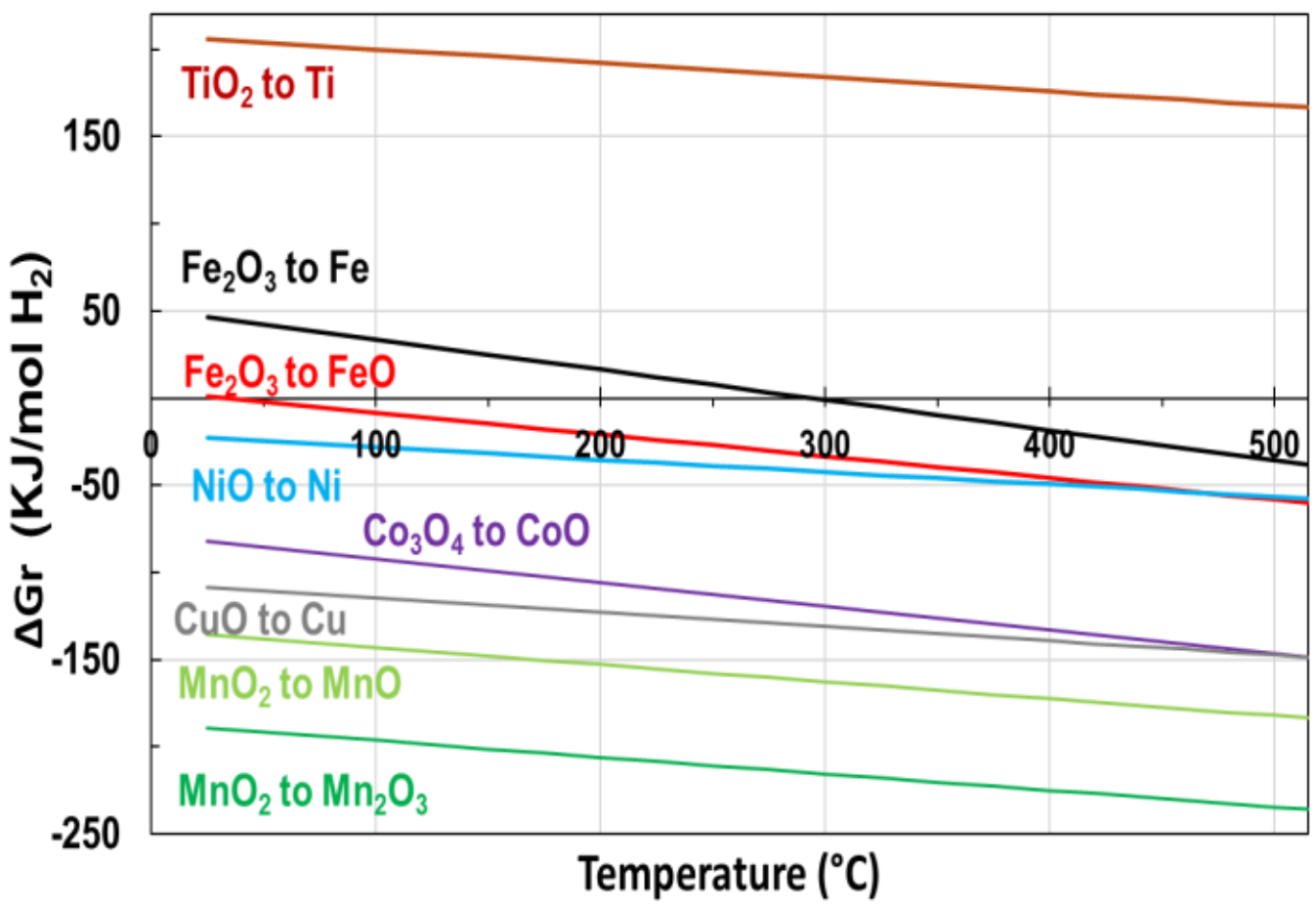

Figure S1: Thermodynamic calculations for Gibbs free energy (ΔG) for various reducible metal-oxides under 5% H_2 gas 0.1% H_2O as a hypothetical reaction condition.

1.4. H_2-Temperature-Programmed Reduction (H_2-TPR)

The H_2-TPR measurements were performed using a Micromeritics AutoChem 2910 instrument. A cold trap was placed in the gas line between the sample tube and the thermal conductivity detector (TCD) to capture any water released during the reduction process. The TCD intensity was calibrated using a standard CuO sample to calculate peak area or H_2 consumption.

A specific amount of finely powdered sample was loaded into a U-shape quartz tube, positioned on top of quartz wool. The sample was re-oxidized *in situ* using UHP O_2 (10 mL/min) by heating from room temperature to 300 °C (10 °C/min) and held for 30 minutes, and cooled to ambient temperature. Once at room temperature, the sample was purged with UHP Ar (30 mL/min) before introducing hydrogen (5 vol. % H_2/Ar, 50 mL/min). The TCD baseline was allowed to stabilize for 30 minutes, followed by heating the sample to 500-600 °C using the desired ramp rate.

Figure S2 shows a comparison between the reduction peak of the reference commercial MnO_2 (black) and the synthesized supported (Blue) and pure unsupported (Grey) MnO_x samples. H_2-TPR measurements on the commercial bulk MnO_2 show a reduction peak at ~450 °C (**Figure S2**). The H_2-TPR peak area indicates the hydrogen uptake is ~1.98 H/Mn (mol/mol).

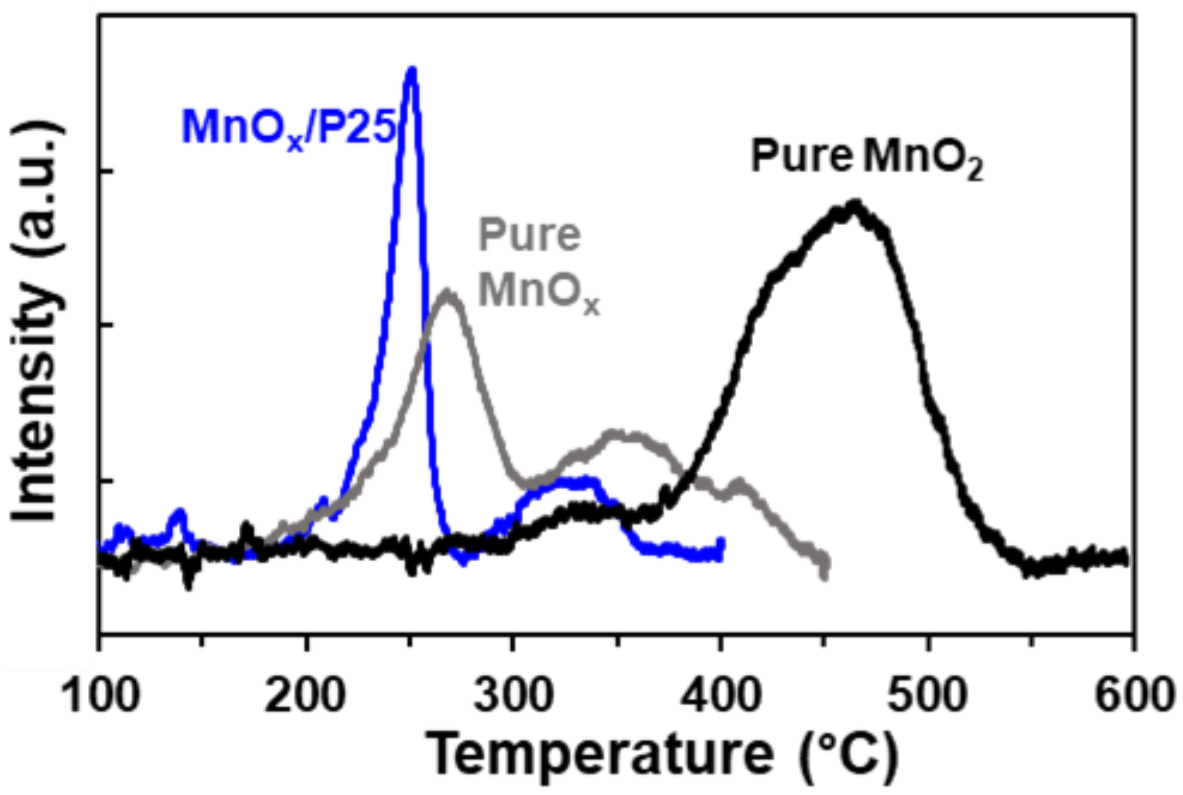

Figure S2: H_2-TPR data measured on different types of MnO_x samples. Blue: $O_xMn/P25$ (300 mg sample with 2% Mn or ~450 Mn /100 nm^2 support), grey: Pure MnO_x (equivalent to 8 mg Mn), and black: Pure reference MnO_2 (equivalent to 15 mg Mn). H_2-TPR performed under 5% H_2/Ar with 5 °C/min ramp rate.

A. H_2-TPR analysis of MnO_x loading effect in presence of 1% Au/P25.

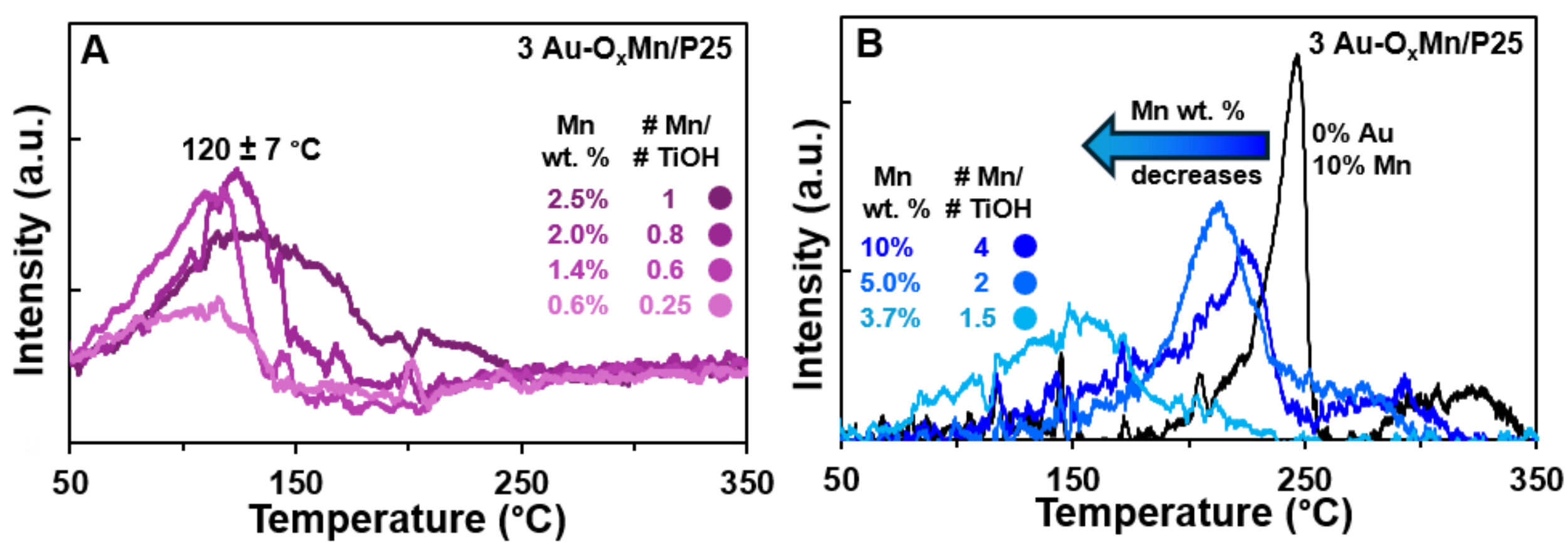

Figure S3: H_2-TPR data measured on 3 Au-$O_xMn/P25$ catalysts with **(A)** low Mn loading (0.6 – 2.5 wt.% Mn – or below one equivalent Mn/TiOH≤1 coverage) and **(B)** high Mn loadings (≥ 3.7% Mn) and constant Au loading (1%wt or 3 Au-MSI sites/100 nm^2). The H_2-TPR experiments were performed under 5% H_2/Ar with a 5 °C/min ramp rate. Black plot presents the Au-free sample (10% Mn/P25)

To summarize, our control tests examining Mn loading effects on the MnO_x reduction temperature (with 0, 0.3, and 3 Au-MSI sites /100 nm^2) show Mn loading has no impact below 3.7 wt. %. This value is >1.5 Mn/TiOH, and suggests the onset formation of less well dispersed MnO_x. Mn surface densities of ~450 Mn /100 nm^2 support and lower were therefore used in all quantitative studies.

B. H_2-TPR analysis of MnO_x/P25 sample mass effect on MnO_x reduction peak max.

The study was conducted on O_xMn_{550}/P25 (2.5 wt.% Mn) at two very different sample mass to examine the error caused by mass such as gas diffusion effects and reproducibility.

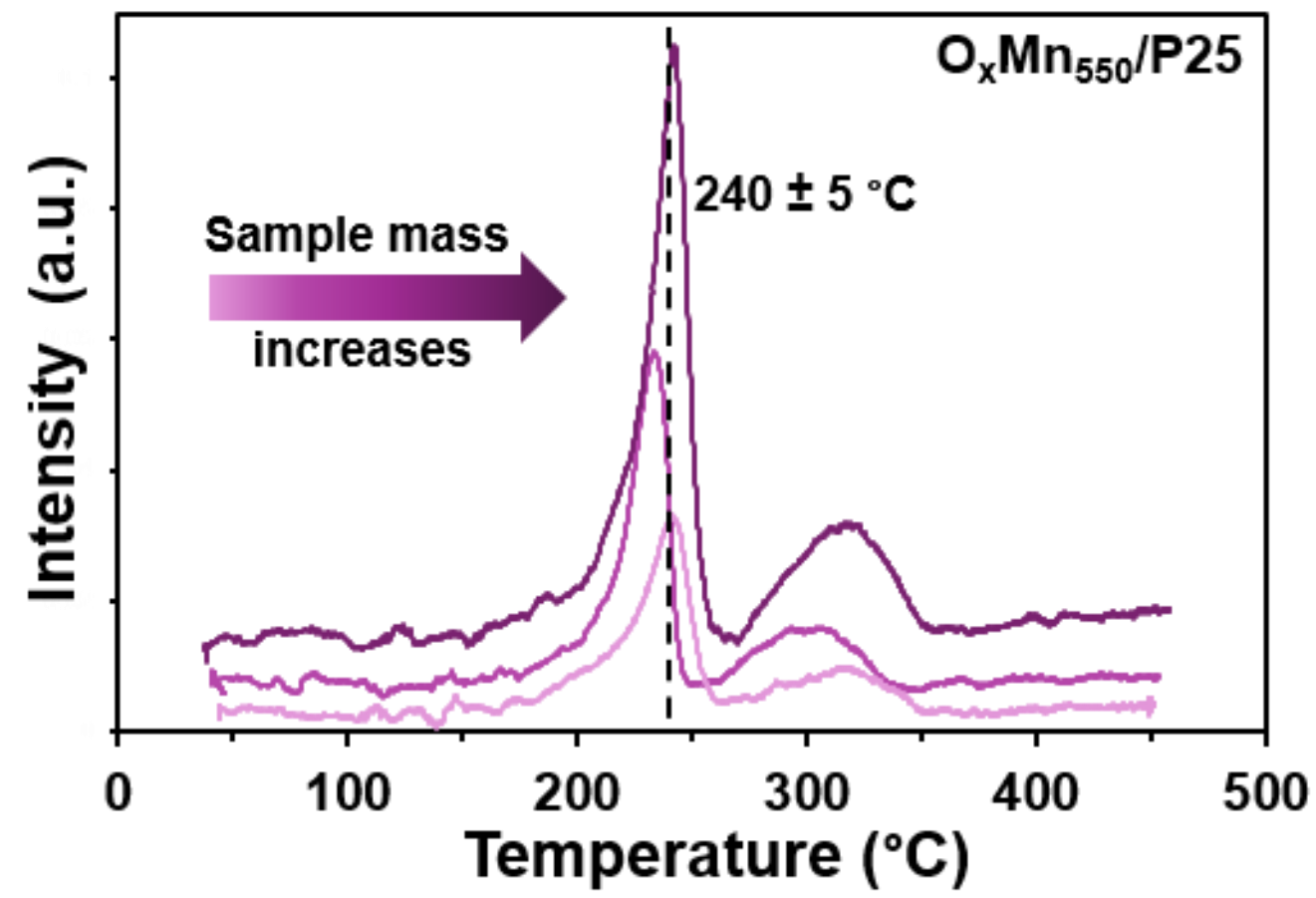

Figure S4: H_2-TPR data measured on O_xMn_{550}/P25 (2.5 wt.% Mn) with different sample masses, 150, 250, and 450 mg. The H_2-TPR experiments were performed under 5% H_2/Ar with a 5 °C/min ramp rate.

C. H_2-TPR analysis of Au loading effects on MnO_x reduction kinetics.

The study was performed in the presence of ~450 Mn /100 nm^2 support and several Au loadings (0, 0.1, 0.2, 0.6, 2 Au-MSI sites/100 nm^2). Both figures show peak max shifts to lower temperatures as Au loading increases for two different ramp rate studies.

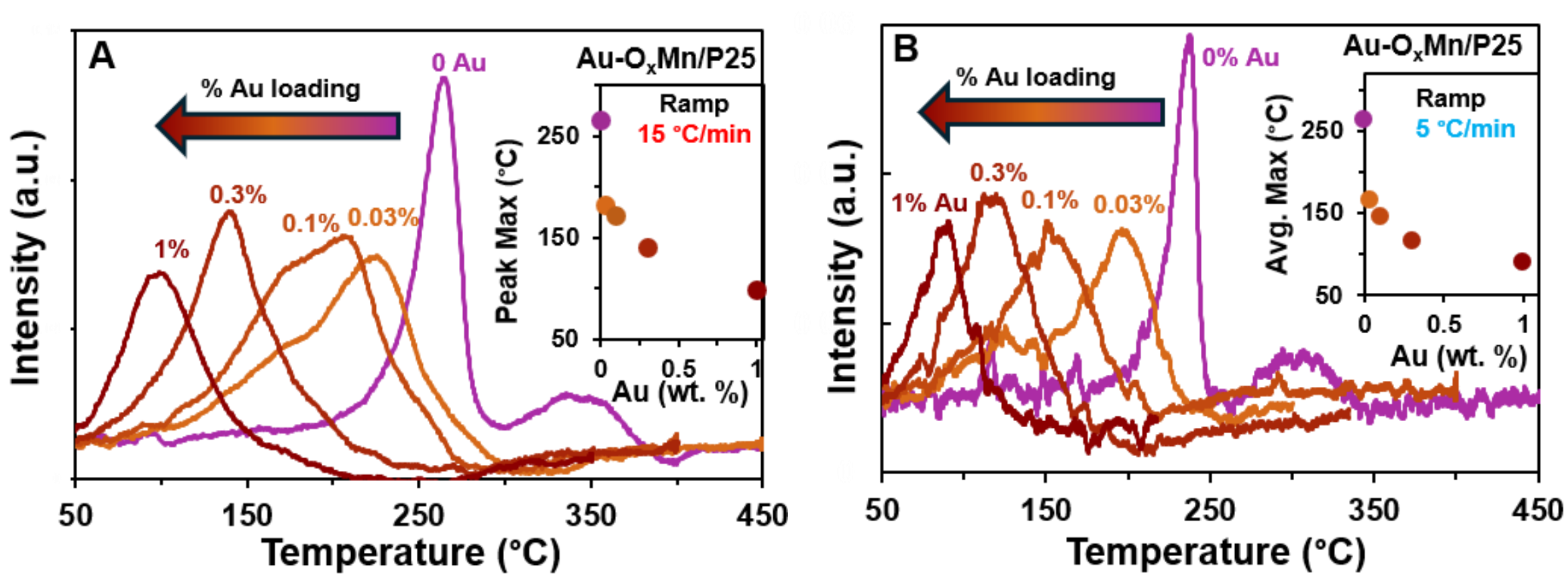

Figure S5: H_2-TPR profiles measured on Au-O_xMn/P25 catalysts with various Au loadings 0, 0.03, 0.1, 0.3, and 1 wt. % Au (corresponding to 0.1, 0.2, 0.6, 2 Au-MSI sites /100 nm^2, respectively) and constant Mn loading (2 wt. % Mn, 450 Mn /100 nm^2 support). The inset plots the center the reduction peak as a function of Au loading. Studies were performed under 5% H_2/Ar with **(A)** 15 °C/min **(B)** 5 °C/min ramp rate.

2. Kissinger analysis of MnO_x reduction E_{app} using H_2-TPR

2.1. H_2-TPR at various ramp rates

The figures below show the H_2-TPR measurement at various ramp rate and Au loadings. The peak max temperature data were used in Kissinger analysis. plot A's represent H consumption rate vs. Time and plot B's represent H consumption rate vs. Temperature.

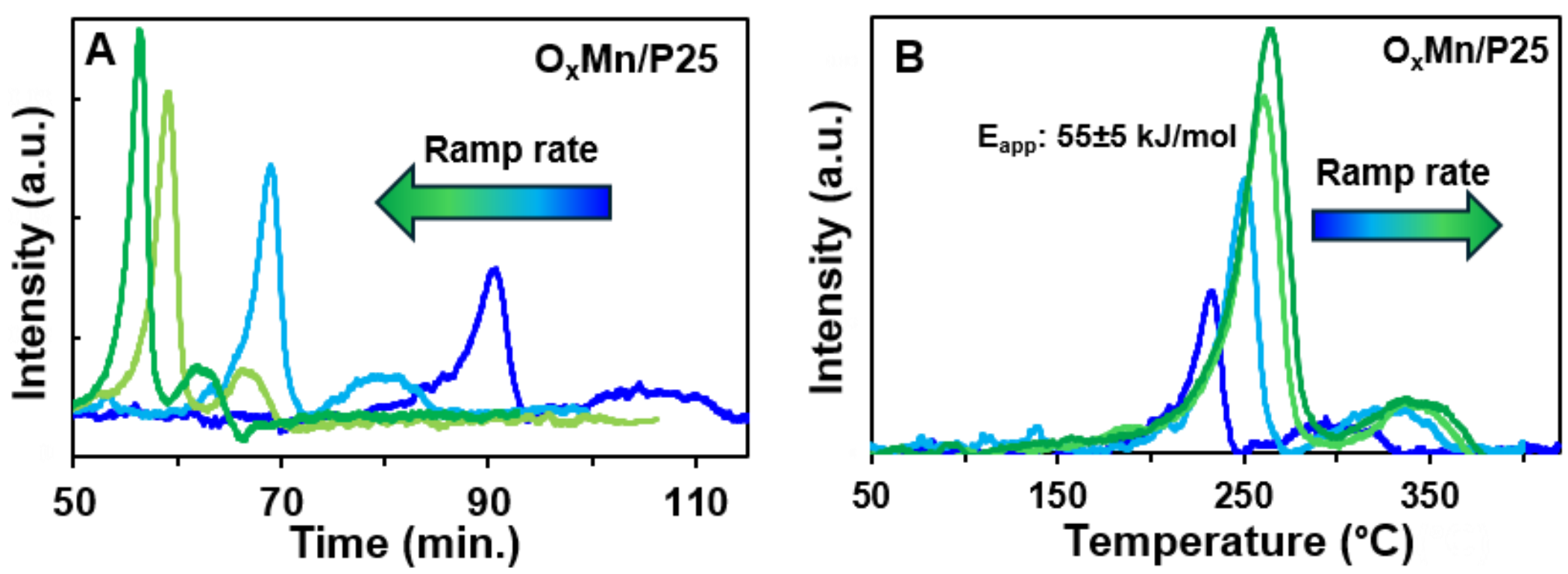

Figure S6. Peak max (T_m) shift vs. A) Temperature and B) Time for O_xMn_{450}/P25 (2 wt. % Mn or ~450 Mn /100 nm^2 support) sample measured by H_2-TPR under 5% H_2/Ar at ramp rates (β): 5- 8- 12- 15 °C/min.

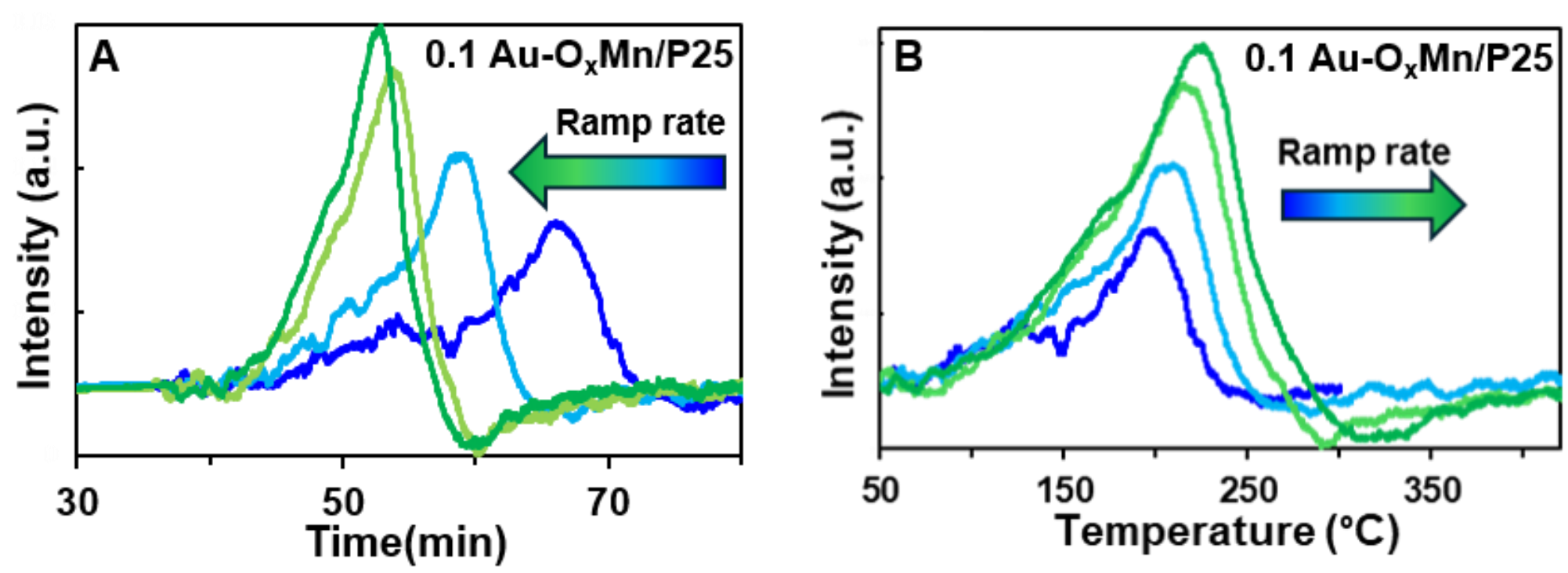

Figure S7. Peak max (T_m) shift vs. A) Temperature and B) Time for 0.1Au-O_xMn_{450}/P25 (0.03 wt. % Au and 2 wt. % Mn) (0.1 Au active sites /100 nm^2 , 450 Mn /100 nm^2 support) sample measured by H_2-TPR under 5% H_2/Ar at ramp rates (β): 5- 8- 12- 15 °C/min.

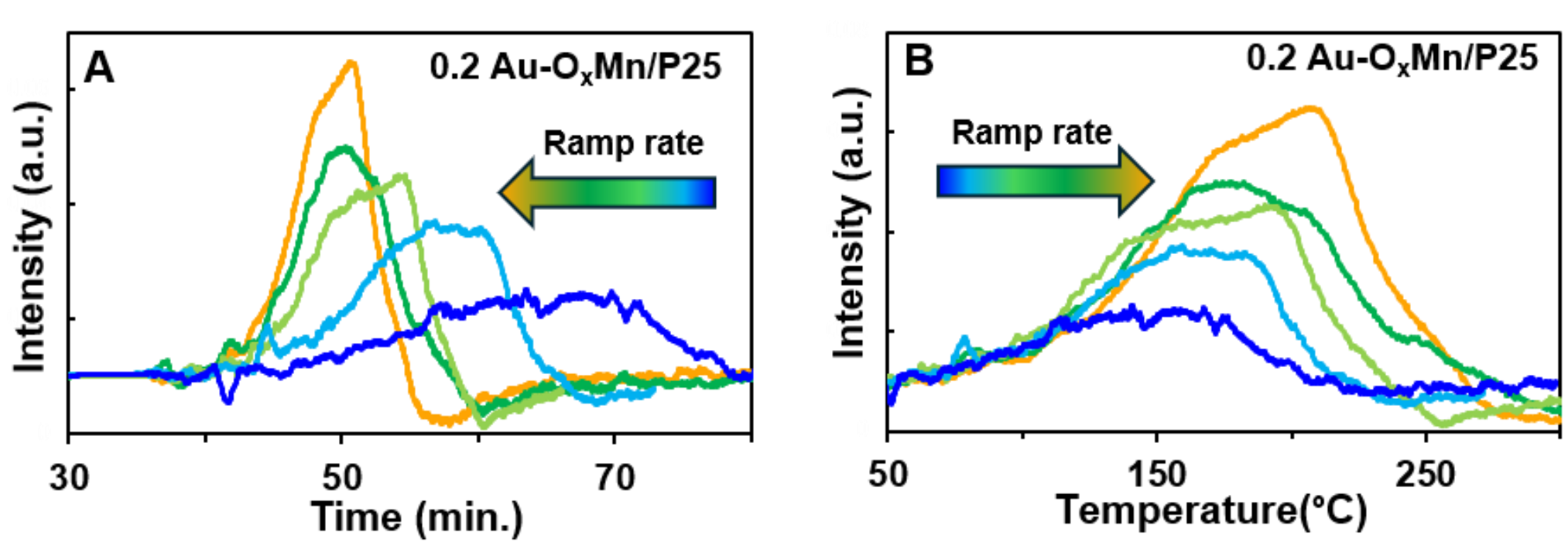

Figure S8. Peak max (T_m) shift vs. A) Temperature and B) Time for 0.2Au-$O_x$$Mn_{450}$/P25 (0.1 wt. % Au , 2 wt. % Mn) (0.2 Au active sites /100 nm^2 , 450 Mn /100 nm^2 support) sample measured by H_2-TPR under 5% H_2/Ar at ramp rates (β): 5- 8- 12- 13.5- 15 °C/min.

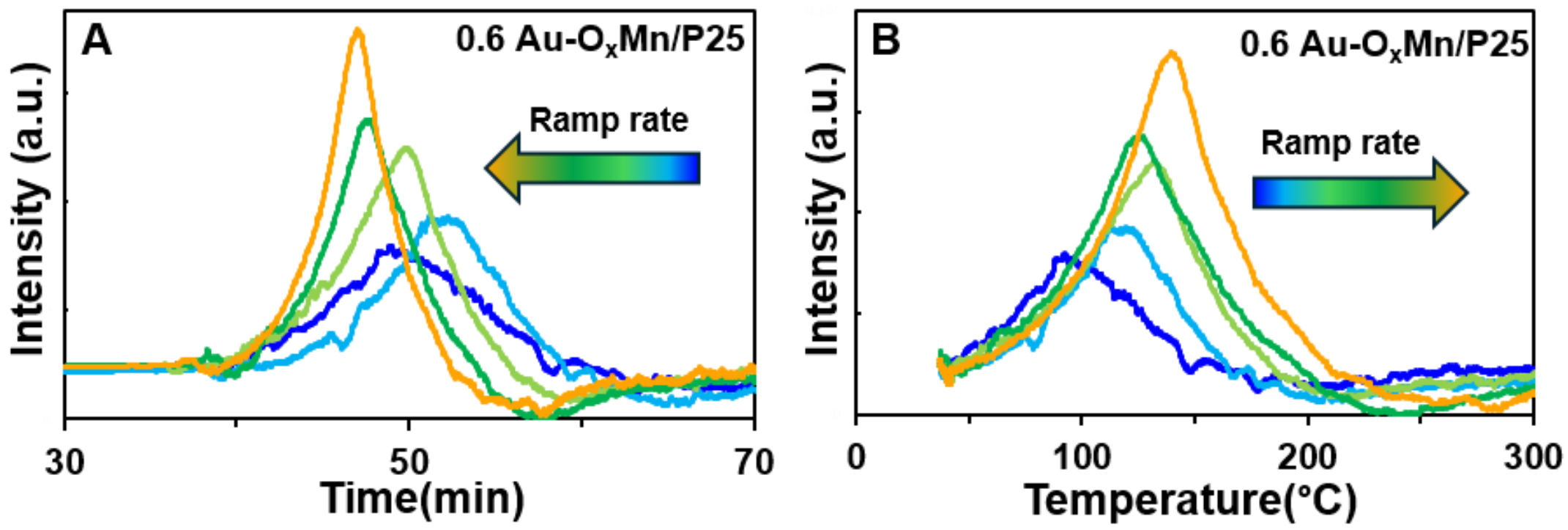

Figure S9. Peak max (T_m) shift vs. A) Temperature and B) Time for 0.6Au-$O_x$$Mn_{450}$/P25 (0.3 wt. % Au, 2 wt. % Mn) (0.6 Au active sites /100 nm^2, 450 Mn /100 nm^2 support) sample measured by H_2-TPR under 5% H_2/Ar at ramp rates (β): 7- 8- 10- 13- 15 °C/min.

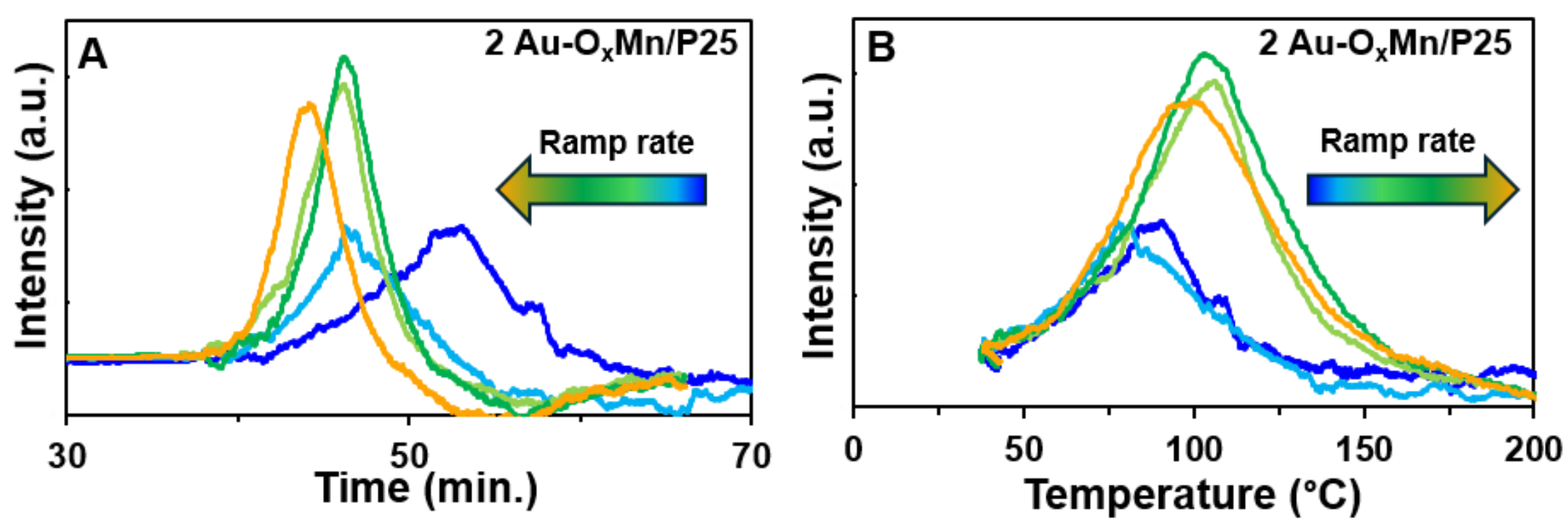

Figure S10. Peak max (T_m) shift vs. A) Temperature and B) Time for 2Au-$O_x$$Mn_{450}$/P25 (1 wt. % Au, 2 wt. % Mn) (2 Au active sites /100 nm^2, 450 Mn /100 nm^2 support) sample measured by H_2-TPR under 5% H_2/Ar at ramp rates (β): 5- 8- 11- 13- 15 °C/min.

2.2. Peak fitting

Figure S11 shows the peak fitting and deconvolution of H_2-TPR traces for low-Au loading samples **A)** 0.1 Au-MSI /100 nm^2 and **B)** 0.2 Au-MSI/100 nm^2. The peak max data were used in Kissinger analysis to determine E_{app}.

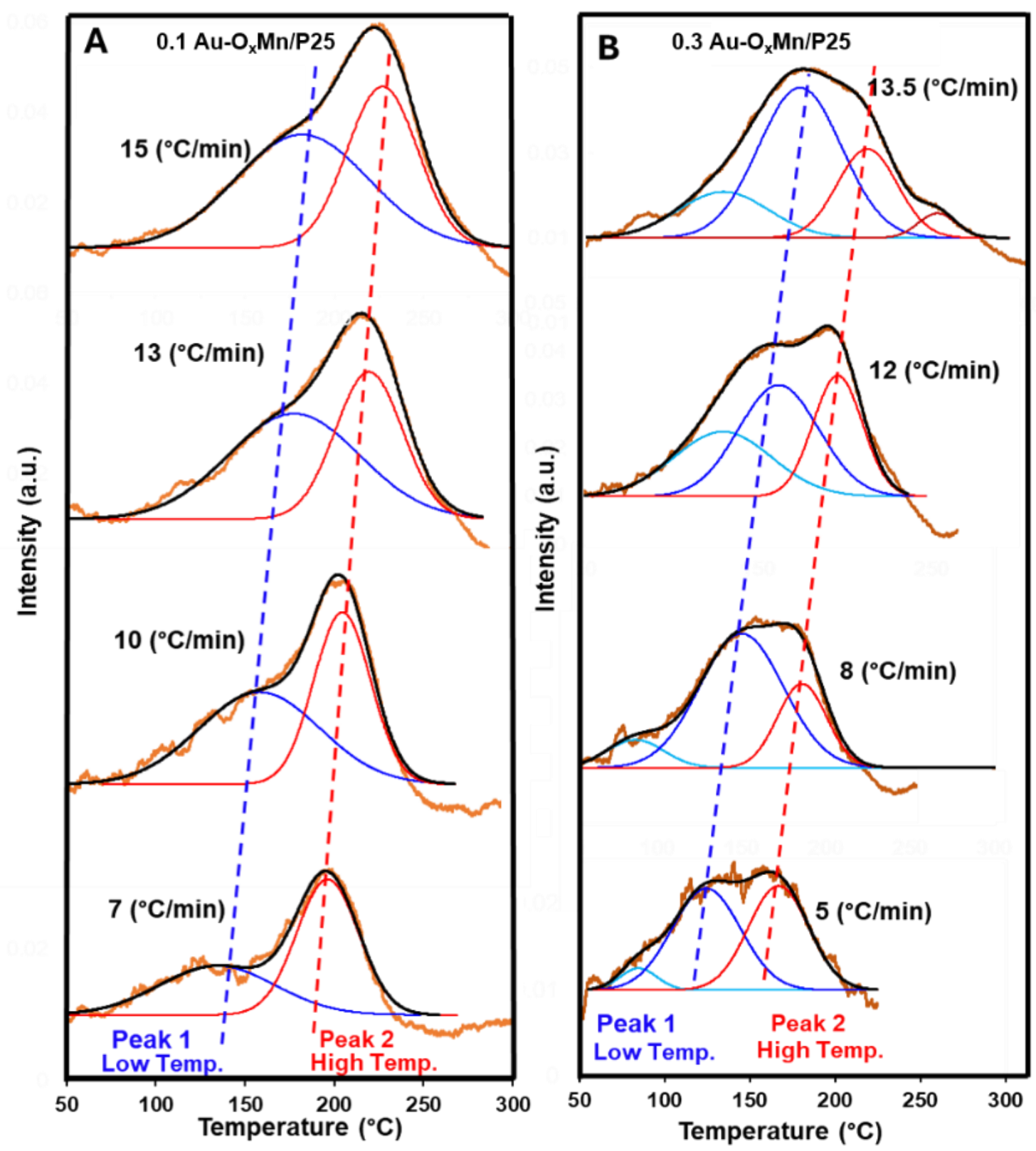

Figure S11. Deconvoluted TPR profiles obtained for Au-O_xMn_{450}/P25 (2 wt. % Mn, 450 Mn /100 nm^2 support) as a function of heating rate (5–15 °C/min)- Au loadings are **(A)** 0.03 wt.% Au (0.1 Au-MSI/100 nm^2) **(B)** 0.1 wt.% Au (0.2 Au-MSI/100 nm^2).

The slope of each plot shows E_{app} measured by Kissinger equation. The Kissinger analysis shows that the Kissinger E_{app} drops as Au loading increases.

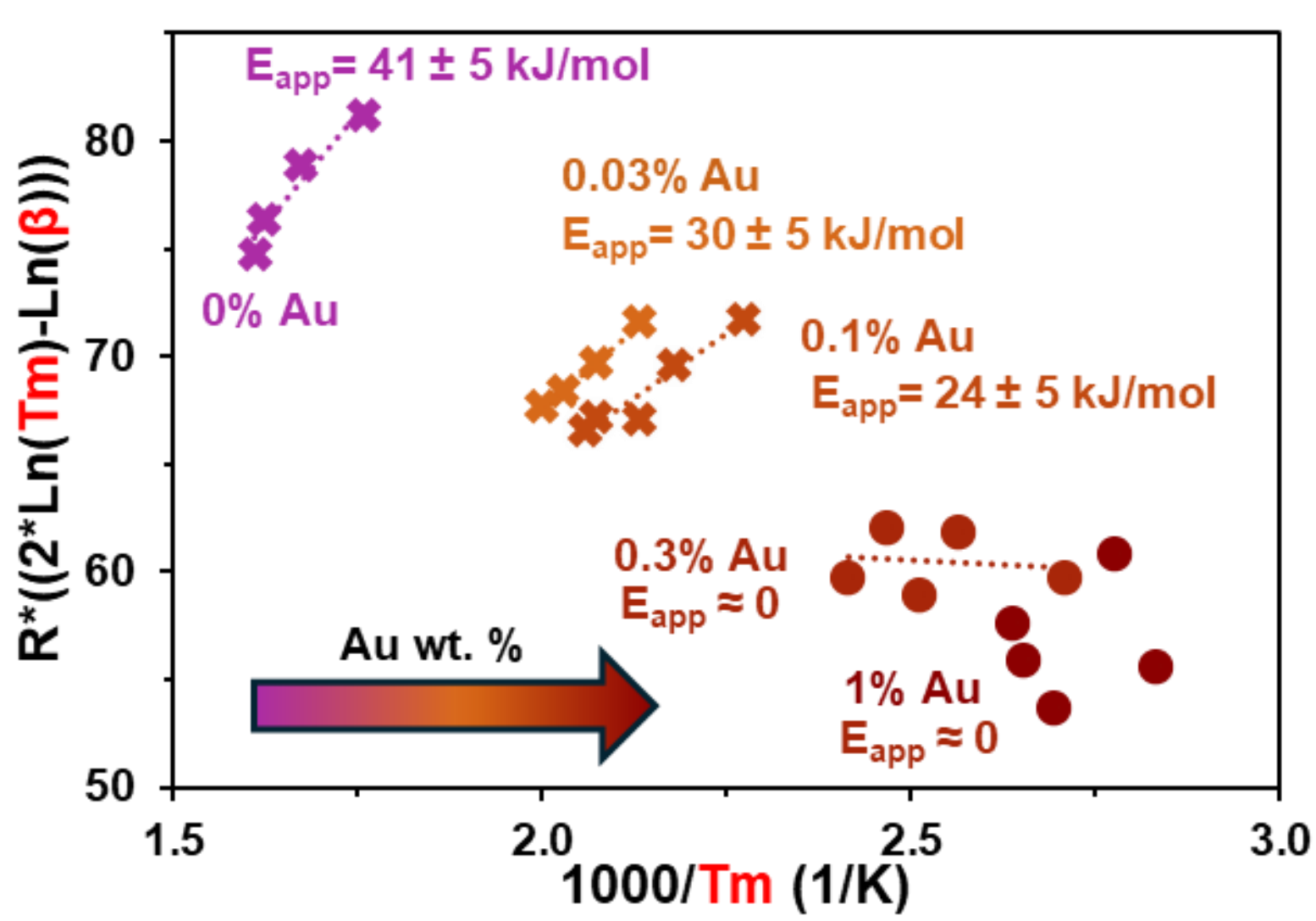

Figure S12. The summary of Kissinger analysis of high temperature peak for Au-MnO_x/P25 catalysts with various Au loading 0, 0.03, 0.1, 0.3, and 1 wt. % Au (corresponding to 0.1, 0.2, 0.6, 2 Au-MSI/100 nm^2 , respectively) and similar 2 wt. % Mn loading or equivalent 450 Mn /100 nm^2 support. The H_2-TPR experiments were performed at various ramp rates of 5-15 °C/min under 5% H_2/Ar.

3. Characterization

3.1. X-ray fluorescence spectroscopy (XRF)

The analysis was performed using Epsilon XRF spectrometer (Malvern Panalytical) and 15 W energy dispersive x-ray fluorescence (EDXRF). With a detector resolution of 135 eV at 5.9 keV/1000 cps. Approximately 200 mg of loose powders were weighed in small mass holders. A 0.6 µm polypropylene film was used to seal sample holders.

Table S2: Elemental analysis for Pt and Pd samples

Catalyst	Metal (Au, Pt, Pd) (wt. %)	Mn (wt. %)	Active sites [a] $\frac{}{100\ nm^2}$
10%Mn/P25	----	9.6± 0.1	----
0.03%Au-2%Mn/P25	0.035 ± 0.001	1.8± 0.01	0.1
0.1%Au-0.6%Mn/P25	0.09 ± 0.001	0.47± 0.05	0.2
0.002%Pt-2.0%Mn/P25	0.0020± 0.0005	1.8 ± 0.1	0.1
0.007%Pt-2.0%Mn/P25	0.0096 ± 0.0005	1.9 ± 0.1	0.3
0.02%Pt-2.0%Mn/P25	0.024 ± 0.001	1.9 ± 0.1	0.9
0.07%Pt-2.0%Mn/P25	0.089 ± 0.001	1.6 ± 0.1	2.9
0.004%Pd-2.0%Mn/P25	0.0039± 0.0005	1.5 ± 0.1	0.3
0.01%Pd-2.0%Mn/P25	0.015 ± 0.001	2.0 ± 0.1	0.9
0.03%Pd-2.0%Mn/P25	0.023 ± 0.001	1.8 ± 0.1	2
0.04%Pd-2.0%Mn/P25	0.038 ± 0.001	1.7 ± 0.1	2.9

[a] the number of metal(Au, Pt, Pd) H_2 activation sites.

*TiO_2 support has less than 1% SiO_2 impurity.

*Major impurities are CaO 300-400 ppm – Cl 200-300 ppm - MgO 200-350 ppm -

3.2. Catalyst Surface Characterization

3.2.A. X-ray photoelectron spectroscopy (XPS)

XPS experiments were performed using a Physical Electronics VersaProbe III instrument equipped with a monochromatic Al kα x-ray source (hν = 1,486.6 eV) and a concentric hemispherical analyzer. Charge neutralization was performed using both low energy electrons (<5 eV) and argon ions. The binding energy axis was calibrated using sputter-cleaned Cu (Cu 2p3/2 = 932.62 eV, Cu 3p3/2 = 75.1 eV) and Au foils (Au 4f7/2 = 83.96 eV). Peaks were charge referenced to CHx band in the carbon 1s spectra at 284.8 eV. Measurements were made at a takeoff angle of 45° with respect to the sample surface plane. This resulted in a typical sampling depth of 3-6 nm (95% of the signal originated from this depth or shallower). Quantification was done using instrumental relative sensitivity factors (RSFs) that account for the x-ray cross section and inelastic mean free path of the electrons. On homogeneous samples major elements (>5 atom%) tend to have standard deviations of <3% while minor elements can be significantly higher. The analysis size was ~200µm in diameter.

The work by M. P. Seah[10] was applied for the corresponding standard values for calibrating the binding energy scales of x-ray photoelectron spectrometer (XPS) tools and analysis.

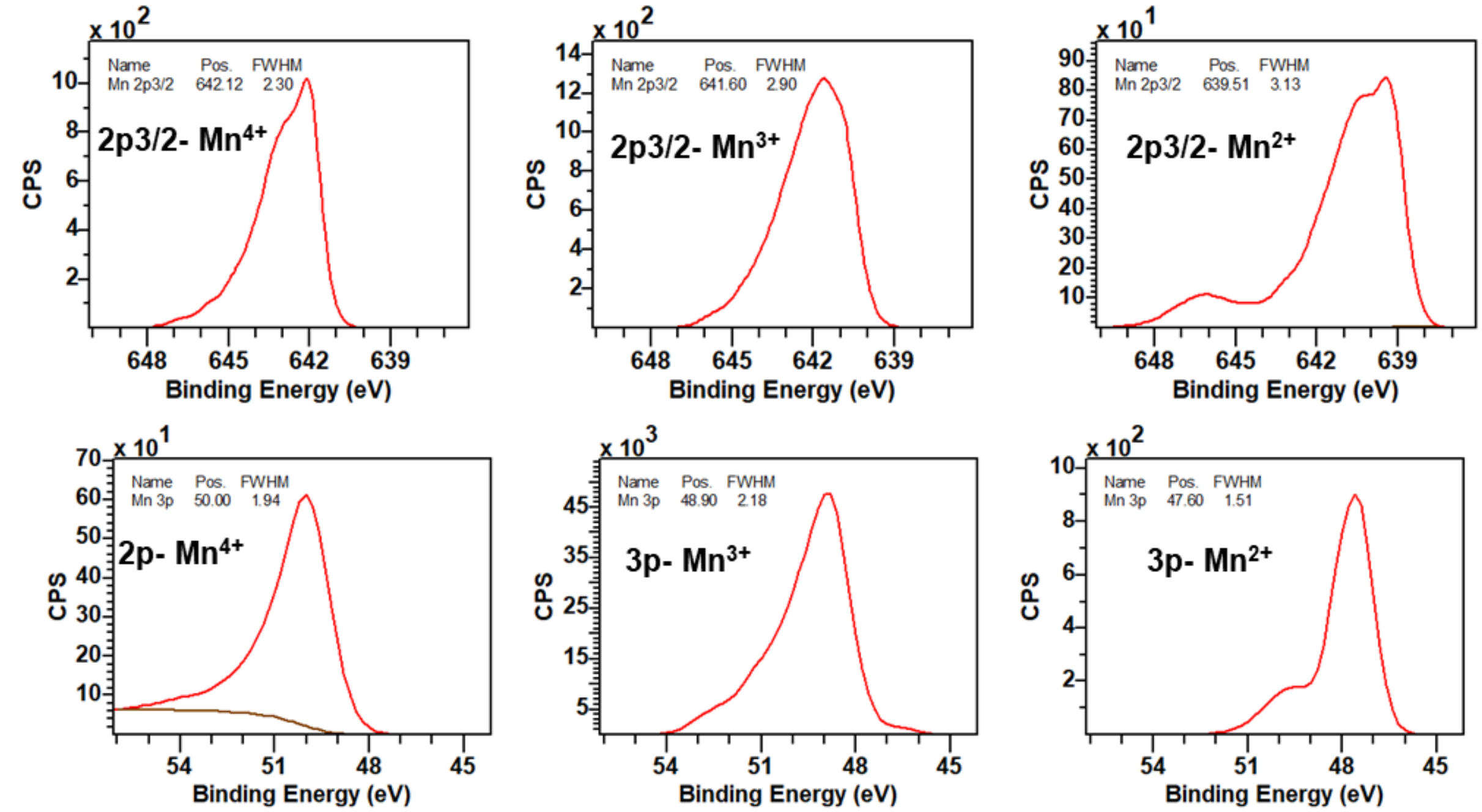

Figure S13 : Synthesized reference 2p3/2 and 3p peaks correspond to Mn^{2+}, Mn^{3+}, and Mn^{4+} applied for peak fitting using the work by Eugene S. Ilton[11].

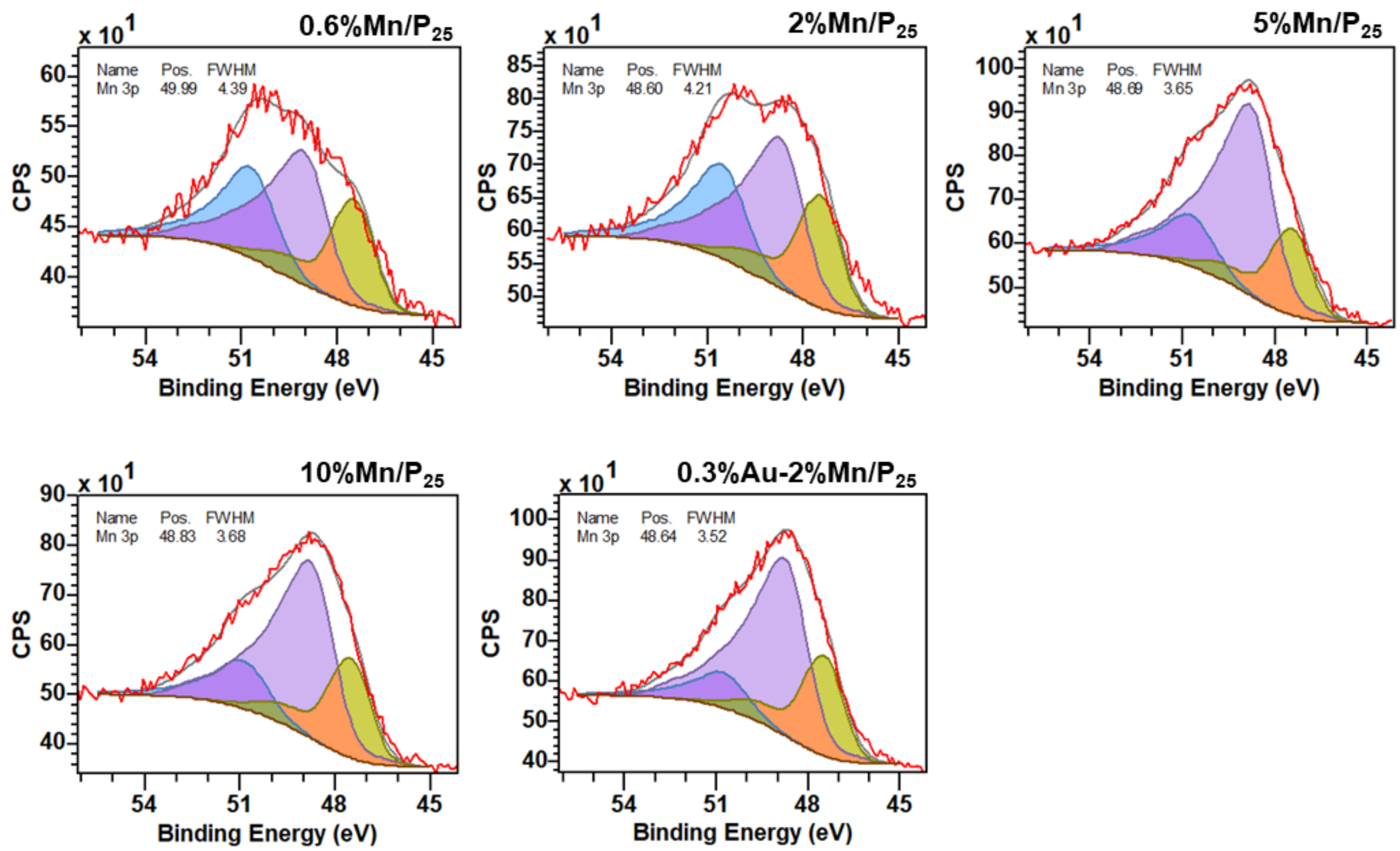

Figure S14: Curve fitting of the Mn 3p spectra with synthesized reference spectra

Interpretation of Manganese chemical oxidation state based on XPS

The manganese oxidation states were assessed with the aid of paper by Ilton *et al* [11]. The Ilton paper examined various reference manganese compounds of known and mixed Mn oxidation states. They used the position and shapes of the Mn 2p, 3s and 3p spectra. The details in that publication were used first to produce synthetic spectra that compared favorably to published Mn spectra from Iltron and other peer reviewed references. Next, these fitting parameters for the 2p and 3p spectra were applied to the data acquired on these samples. [The Mn 3s was too weak and was interfered with by Au 4f on one sample.

The samples contained Ti^{4+}, Mn, O and C. One sample contained a small amount of Au. Peak fitting suggested the presence of Mn^{2+} and Mn^{3+} and Mn^{4+} on all samples. The Mn spectral line shapes for the reference pure compounds are complex and asymmetric which makes it hard to certainly determine the oxidation state. Moreover, the presence of Surface hydroxide might affect the oxidation state of the surface MnO_x due to the presence of O atoms on TiO_2 , it was impossible to use O peak position to determine the oxidation state. The center of mass and overall shape of the Mn spectra on these samples were most consistent with $Mn^{3+,4+}$.

Table S3: The summary of XPS analysis data of samples before and after reduction in H_2-TPR using Mn 3p peak fitting.

**Samples**	**Mn% extracted from XPS data**			**Avg. oxidation state**
	Mn 3p fitting			XPS- 3p fitting
	Mn^{2+}	Mn^{3+}	Mn^{4+}	
O_xMn_{140}/P25	27	45	28	3.0
O_xMn_{450}/P25	26	47	27	3.0
O_xMn_{1120}/P25 **(Before TPR)**	21	62	18	3.0
O_xMn_{1120}/P25 **(After TPR)**	50	40	10	2.5
O_xMn_{2230}/P25	25	58	17	2.9
0.6Au-O_xMn_{450}/P25	26	62	12	2.9

3.2.B X-ray diffraction analysis (XRD)

Powder X-Ray Diffraction patterns were collected using a Malvern PANalytical Empyrean fitted with a Cu Kα source. A 1Der X-ray detector was used to mitigate signal from Mn fluorescence under the Cu Kα source. Experimental scans were conducted from 20 to 80 °2θ. The reference patterns shown were sourced from the International Crystal Structure Database (ICSD), and their PDF reference numbers are provided.

Anatase: PDF# 01-071-1166

Rutile: PDF# 01-071-0650

MnO_2: PDF# 01-090-9047

The peak around 2θ =27.8°, 43°, and 72° are not visible for 2 and 2.5 wt.% Mn and only show up slightly in 10 wt.% Mn on TiO_2 (O_xMn_{2230}/P25) sample.

Powder XRD patterns show MnO_x particle sizes are very small and the synthesis protocol makes a very dispersed MnO_x particles on P25.

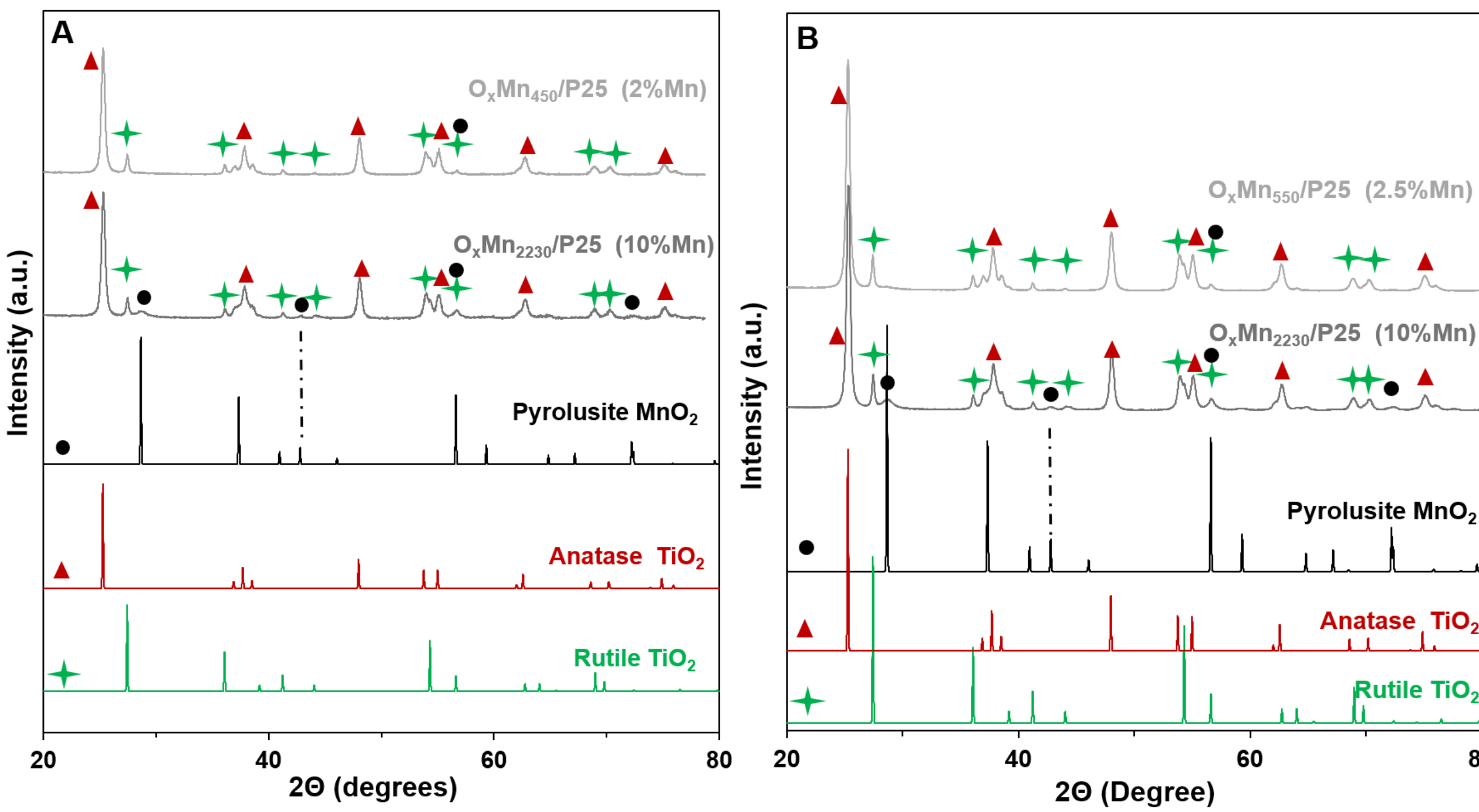

Fig S15: XRD patterns of freshly calcined A) O_xMn_{450}/P25 and O_xMn_{2230}/P25, and B) O_xMn_{550}/P25 and O_xMn_{2230}/P25 samples, repeated with higher resolution. The reference rutile (green), anatase (red), and MnO_2 (black).

Scherrer Crystallite Size for O_xMn_{2230}/P25 (10% Mn/P25) :

Equation: $\tau = \frac{K\lambda}{\beta Cos(\theta)}$ (S4)

τ is crystallite size

K is a shape factor (0.9)

λ is source wavelength (1.54 Å)

β is FWHM (in radians)

θ is Bragg angle (in radians)

First peak crystallite size: β = 1.090°, 2θ = 28.732°

$$\tau = \frac{K\lambda}{\beta Cos(\theta)} = \frac{0.9(1.54\ \text{Å})}{0.0190\ cos(0.251)} = 7.5\ \pm 5\ nm \quad \text{(S5)}$$

3.2.C. HAADF-STEM and EDS Element Mapping

HAADF-STEM:

Metal particle sizes were determined by imaging with scanning/transmission electron microscopy (STEM) using a Talos F200C microscope at an accelerating voltage of 200 kV. To prepare each sample, a small quantity of catalyst was crushed in an agate mortar and pestle and dispersed in isopropanol. A Cu TEM grid (Ted Pella, Inc.) was dipped into this suspension and allowed to dry before analysis. The average Au, Pd, or Pt nanoparticle diameter was determined using ImageJ from the measurement of at least 100 particles (**Figure S16-S18**).

EDS mapping:

High-angle annular dark field scanning transmission electron microscopy (HAADF-STEM) images and energy dispersive X-ray spectroscopy (EDS) elemental maps were collected on the same Talos F200X2 microscope at an accelerating voltage of 200 kV. STEM-EDS elemental maps were analyzed using Velox 3.6.0 software (Thermo Fisher), and figures show net elemental map data. Elemental quantification was performed by spectrum integration across the selected particle region in each map.

2Au–$O_x$$Mn_{450}$/$P_{25}$ (1 wt.% Au, 2 wt. % Mn) :

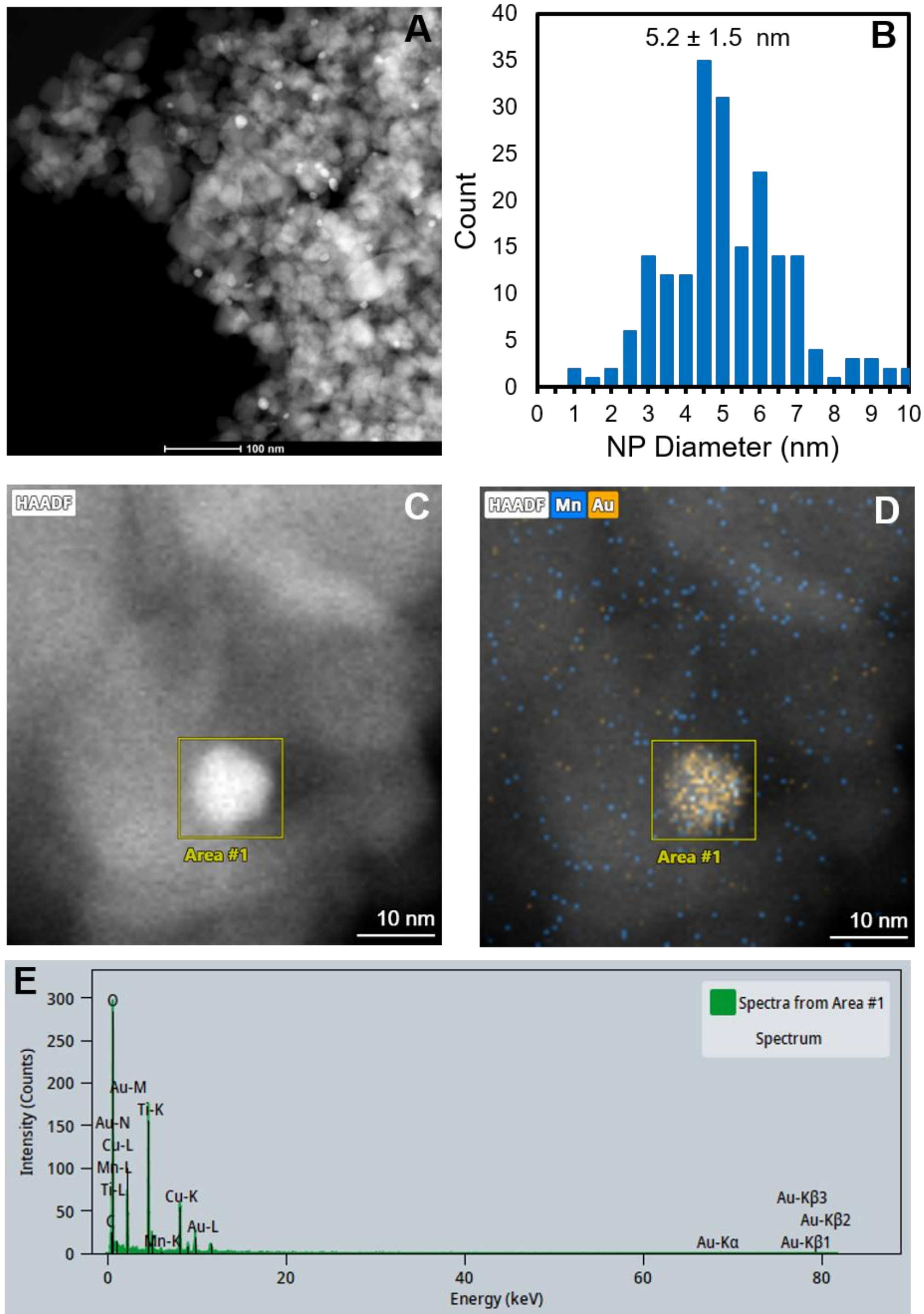

Figure S16: Representative **(A)** STEM-HAADF image and **(B)** corresponding particle-size distributions of freshly calcined $2Au–O_xMn_{450}/P_{25}$ (1 wt.% Au, 2 wt. % Mn), and **(C, D)** Representative EDS mapping images showing Au (yellow-bright) nanoparticles on TiO_2 (gray), MnO_x (blue) nanoparticles on TiO_2. The bright spots correspond to Au; MnO_x particles are not readily distinguishable from the TiO_2 support in STEM due to the similar atomic numbers of Mn (Z = 25) and Ti (Z = 22), which provide insufficient Z-contrast. **(E)** Representative EDS spectrum acquired from the mapped region during EDS elemental mapping, confirming the presence of Au, Ti, and O. Cu peaks originate from the TEM support grid.

Table S4 : Quantitative EDS analysis of the mapped region **(Figure S16)**, showing the atomic and mass fractions of Ti, Mn, and Au obtained from the representative EDS spectrum.

Z	Element	Family	Atomic Fraction (%)	Atomic Error (%)	Mass Fraction (%)	Mass Error (%)	Fit Error (%)
22	Ti	K	87.19	1.17	63.45	2.5	0.76
25	Mn	K	0.00	0.12	0.00	0.12	0.00
79	Au	L	11.97	1.16	35.84	2.52	2.2

0.6Au–$O_x$$Mn_{450}$/$P_{25}$ (0.3 wt.% Au, 2 wt. % Mn) :

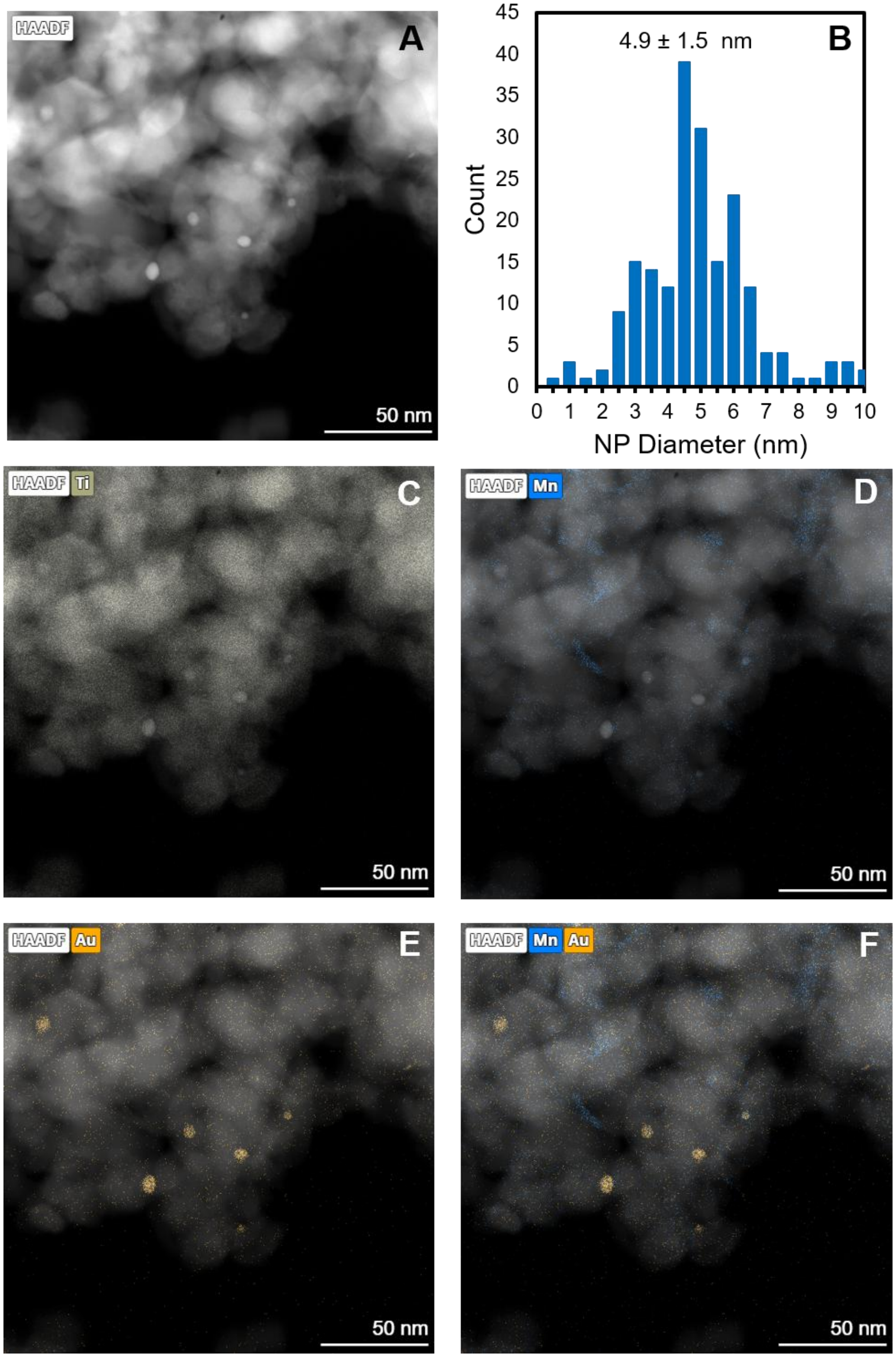

Figure S17: Representative **(A)** STEM-HAADF image and **(B)** corresponding particle-size distributions of freshly calcined 0.6Au–O_xMn_{450}/P_{25} (0.3 wt.% Au, 2 wt. % Mn), and **(C, D, E, F)** Representative EDS mapping images showing Au (yellow-bright) nanoparticles on TiO_2 (gray), MnO_x (blue) nanoparticles on TiO_2. The bright spots correspond to Au; MnO_x particles are not readily distinguishable from the TiO_2 support in STEM due to the similar atomic numbers of Mn (Z = 25) and Ti (Z = 22), which provide insufficient Z-contrast.

3Pt–O_xMn_{450}/P25 (0.073 wt.% Pt, 2 wt. % Mn) :

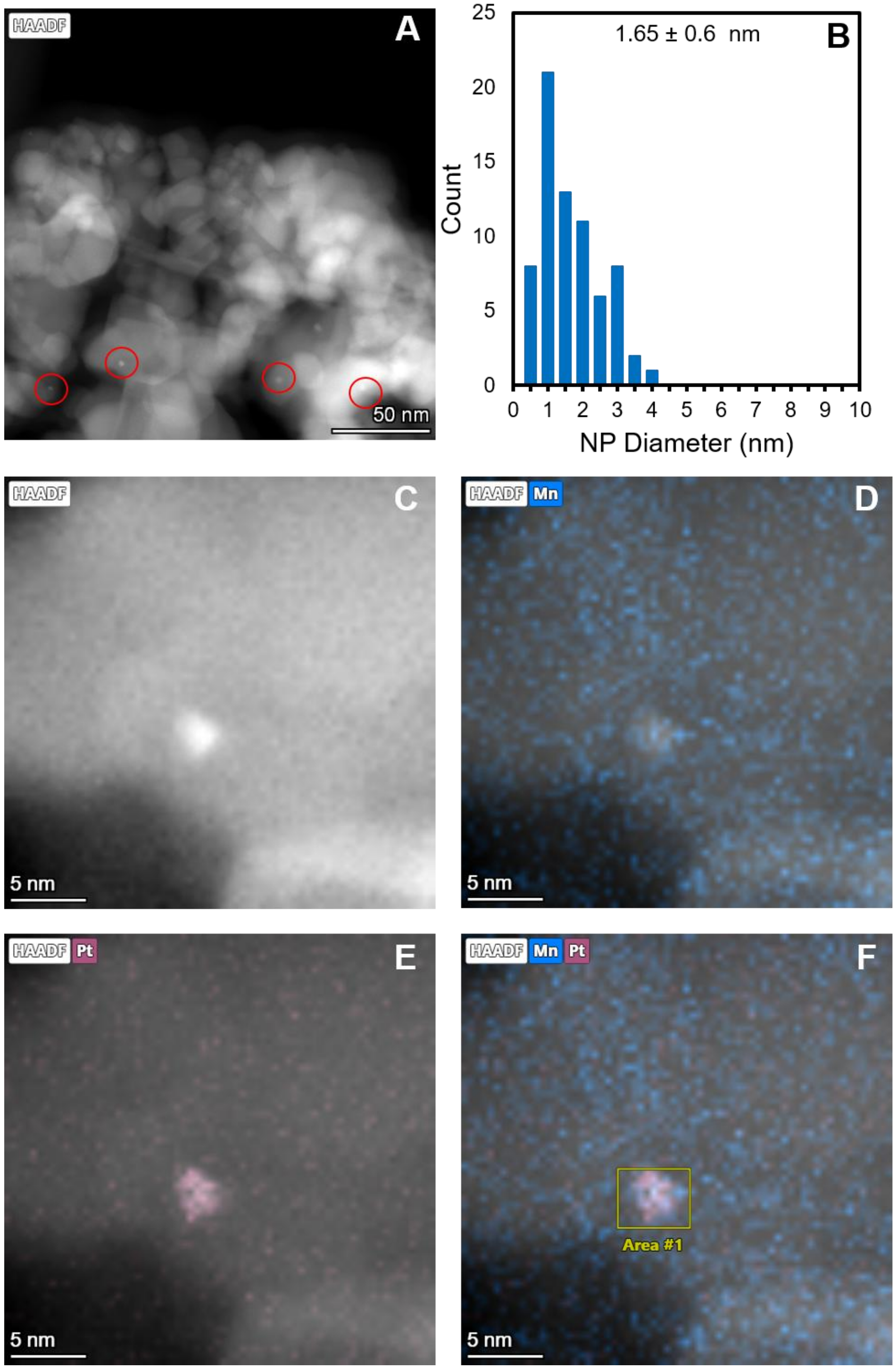

Figure S18: Representative **(A)** STEM-HAADF image and **(B)** corresponding particle-size distributions of freshly calcined 3Pt–O_xMn_{450}/P25 (0.073 wt.% Pt, 2 wt. % Mn), and **(C, D, E, F)** Representative EDS mapping images showing Pt (red-bright) nanoparticles on TiO_2 (gray), MnO_x

(blue) nanoparticles on TiO_2. The bright spots correspond to Pt; MnO_x particles are not readily distinguishable from the TiO_2 support in STEM due to the similar atomic numbers of Mn (Z = 25) and Ti (Z = 22), which provide insufficient Z-contrast.

3.2.D. Au and Pt Active Site Analysis

Au Perimeter and Surface Site Analysis:

The fraction of metal atoms on the surface (%S) or perimeter (%P) of the nanoparticles was determined using previously reported methods modeling Au and Pt particles as cuboctahedra.[3] Perimeter site density (M_{per}), surface site density (M_{surf}), %P, and %S were calculated using the equations S6-S9.

$$\%P = \frac{24 m_M}{5 D_p^2 r_M \rho_M \sqrt{2}} \times 100 \quad \text{(S6)}$$

$$\%S = \frac{12 m_M (3+\sqrt{3})}{5\pi D_p r_M^2 \rho_M \sqrt{2}} \times 100 \quad \text{(S7)}$$

$$M_{per}\left(\frac{\mu mol}{g\ cat.}\right) = \frac{\%M \times \%Per \times 10^6}{M_w(M)} \quad \text{(S8)}$$

$$M_{sur}\left(\frac{\mu mol}{g\ cat.}\right) = \frac{\%M \times \%Sur \times 10^6}{M_w(M)} \quad \text{(S9)}$$

Where:

- m_M is the mass of a single metal atom (3.27×10^{-25} kg/Au atom, 3.24×10^{-25} kg/Pt atom)
- d_p is the circumsphere diameter of a cuboctahedral particle (from TEM)
- r_M is the atomic radius of Au (1.66×10^{-10} m/atom) or Pt (1.39×10^{-10} m/atom)
- ρ_M is the bulk density of Au (19300 kg/m^3) or Pt (21450 kg/m^3)

Calculating %P using mean nanoparticle diameter from a wide distribution of particle sizes leads to significant error due to the non-linear relationship between %P and D_p. Instead, %P was calculated for the entire particle size histogram, considering more than 300 nanoparticles. For perimeter site density determination, bin sizes of 0.25 nm were used, calculating the %P based on the center of each bin. These values were then multiplied by their respective bin count and averaged to estimate the weighted average %P of the samples shown in **Table S2**. The same process was followed to calculate weighted average %S of each sample. Further derivations and justifications pertaining to this analysis can be found in the SI of our previous reports.[3,4] While the average dNP is listed (and commonly used) for Au samples, the distribution of NP diameters (**Figure Hist**) has a significant impact on %S calculations. For example, while Au/P25 and Au/ana have similar dNP averages, Au/P25 has significantly more NPs with $d \leq 2$ nm. Because %S is proportional to 1/diameter^2, these small NPs contribute more to the number of surface sites than larger NPs.

Also, for cubo-octahedral model, the website below is useful:

https://rechneronline.de/pi/cuboctahedron.php.

By assuming a hemispherical particle geometry for Pt the dispersion (70 %) for 1.7 nm average particle size was calculated using the equation S5:

$$D \approx \frac{112}{d(1.7\ nm)} = 65\% \quad (S10)$$

Table S5: Average nanoparticle size, surface metal (M_{surf}) distribution, and perimeter metal (M_{per}) distribution. Uncertainties are calculated from standard deviations.

Catalyst	Average d_{NP} (nm)	%S	%P
0.3-1% Au/TiO_2	5 ± 1.0	38	3

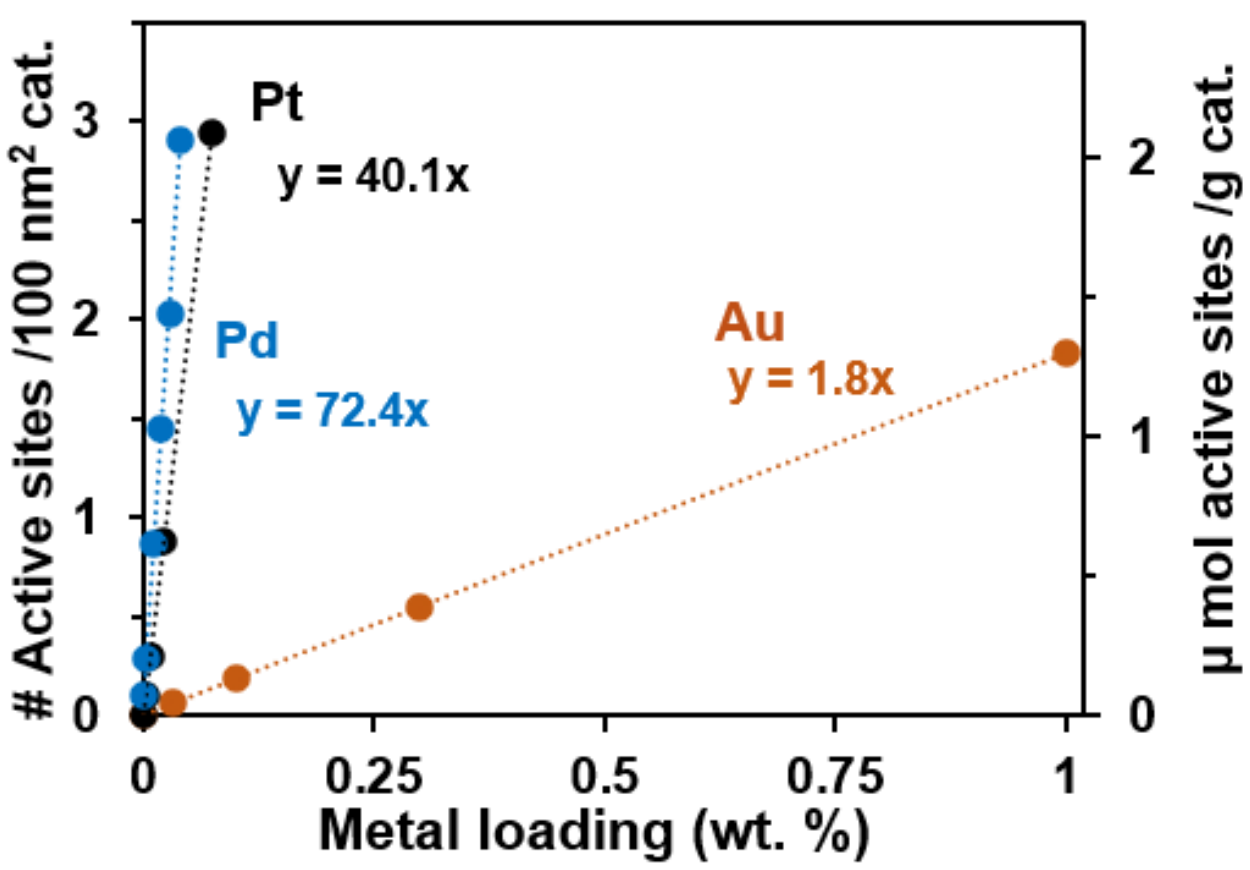

Figure S19: Active metals loadings versus the calculated number of active sites. Conversion plot for Metal loading (wt. %) and the density of active sites for Pd, Pt, and Au catalysts. The number of active sites is normalized per 100 nm² of catalyst surface (left axis) and mass of the samples (right). Active metals loadings versus the calculated number of active sites. The Pt and the Au average particle sizes are 1.7 and 5 nm, respectively.

3.3. UV-Vis spectroscopy

Determination of Optical Band Gap via Tauc Plot:

Diffuse Reflectance UV-Vis spectroscopy (DRS) was conducted using Praying Mantis™ (Harrick Scientific Products) spectrometer. The Praying Mantis™ (DRP) accessory is specifically designed for examining powders by diffuse reflection spectroscopy. The sample was loaded into a sample cup diluted with KBr (non-absorbing powder). The same non-absorbing powder was used to collect the background spectrum. The forbidden band (Eg) values were determined using the modified Kubelka-Munk equation. UV–Vis absorbance spectra were converted to photon energy (hν) using:

hν (eV) = 1240/λ or, (S11)

hν (eV) = 6.242 * (10^18) * hν(J) = 6.242 * (10^18) * 6.626 * (10^-34) * ν, (S12)

where ν is frequency ν = 3 * (10^17)/A (A is absorbance), λ is the wavelength in nm, h is Planck's constant. The Tauc function was approximated using absorbance values, since the sample was fine powder and thickness was unknown. Moreover, the Kubelka–Munk function, *F*(*R*), can be considered proportional to the absorption of radiation[12]. Specifically, $(Abs\cdot h\nu)^n$ was plotted against hν (eV), where n corresponds to the transition type (e.g., n=2 for direct [13],[14] and n=0.5 for indirect[15],[16] transitions). The optical band gap was determined by extrapolating the linear region of the plot to the photon energy hν (eV.) axis. This approach provides a relative estimate of the band gap energy (E_g).

$\alpha(h\nu) = A\,(h\nu - E_g)^n$, A is a constant. E_g is the bandgap of the material. (S13)

n = 2 for an indirect allowed transition (plotted as $(\alpha\, h\nu)^{1/2}$ versus hν);

$\alpha \propto A \Rightarrow$ so $(\alpha h\nu)^n \propto (A h\nu)^n$

Figure S20 shows calcined fresh MnO_x/P_{25} samples have higher light absorbance in visible region than reduced samples.

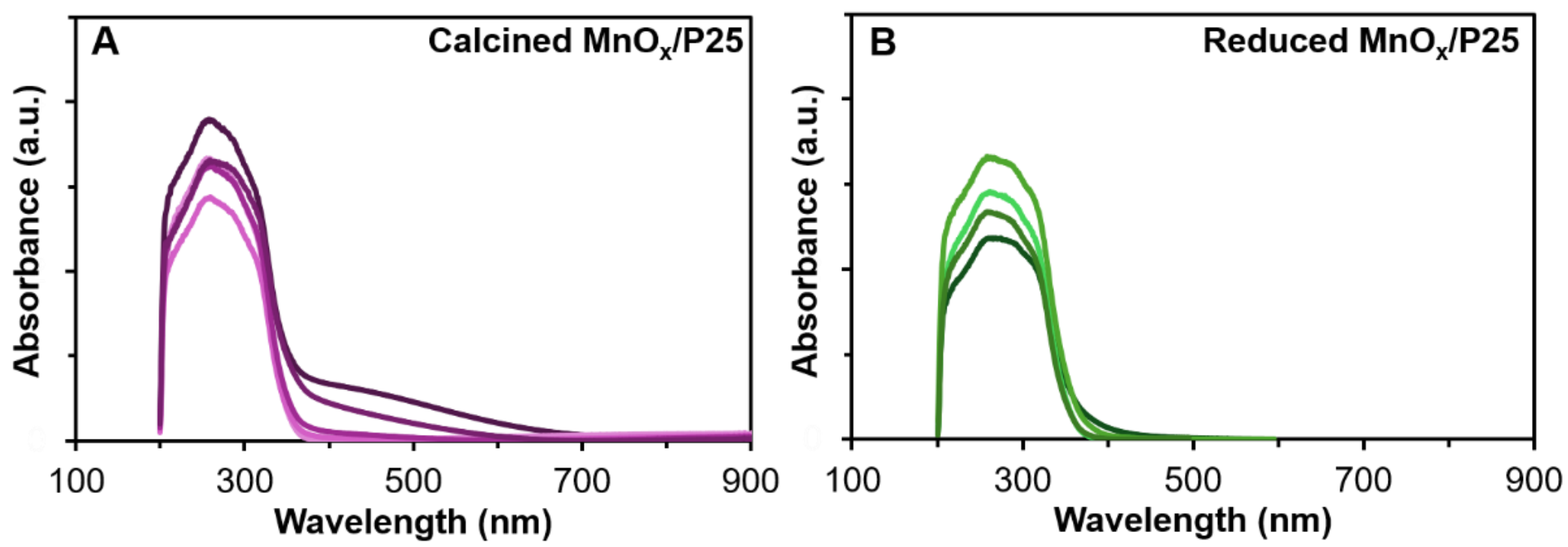

Figure S20: Ultraviolet/visible **(**UV-Vis) absorbance spectroscopy data collected on **(A)** Fresh calcined MnO_x/P25 samples **(B)** Reduced MnO_x/P25 (MnO/P25) samples diluted with KBr. KBr background collected right before the tests. Reduction was performed under 5% H_2/Ar at 400 °C (same as the TPR condition).

The UV–Vis results (**Figure S21**) show that the reduced bulk MnO_x has a larger band gap than the fresh MnO_x sample. Before H_2-TPR, the band gap was approximately 1.5 eV, whereas after H_2-TPR it increased to about 3.5 eV. These values are consistent with the reported reference band gaps of MnO_2 (Mn^{4+}, ~1.2 ± 0.3 eV)[15, 17] and MnO (Mn^{2+}, ~3.7 ± 0.3 eV).[18, 19] Overall, the data indicate that bulk MnO_2 was reduced to MnO at approximately 400 °C.

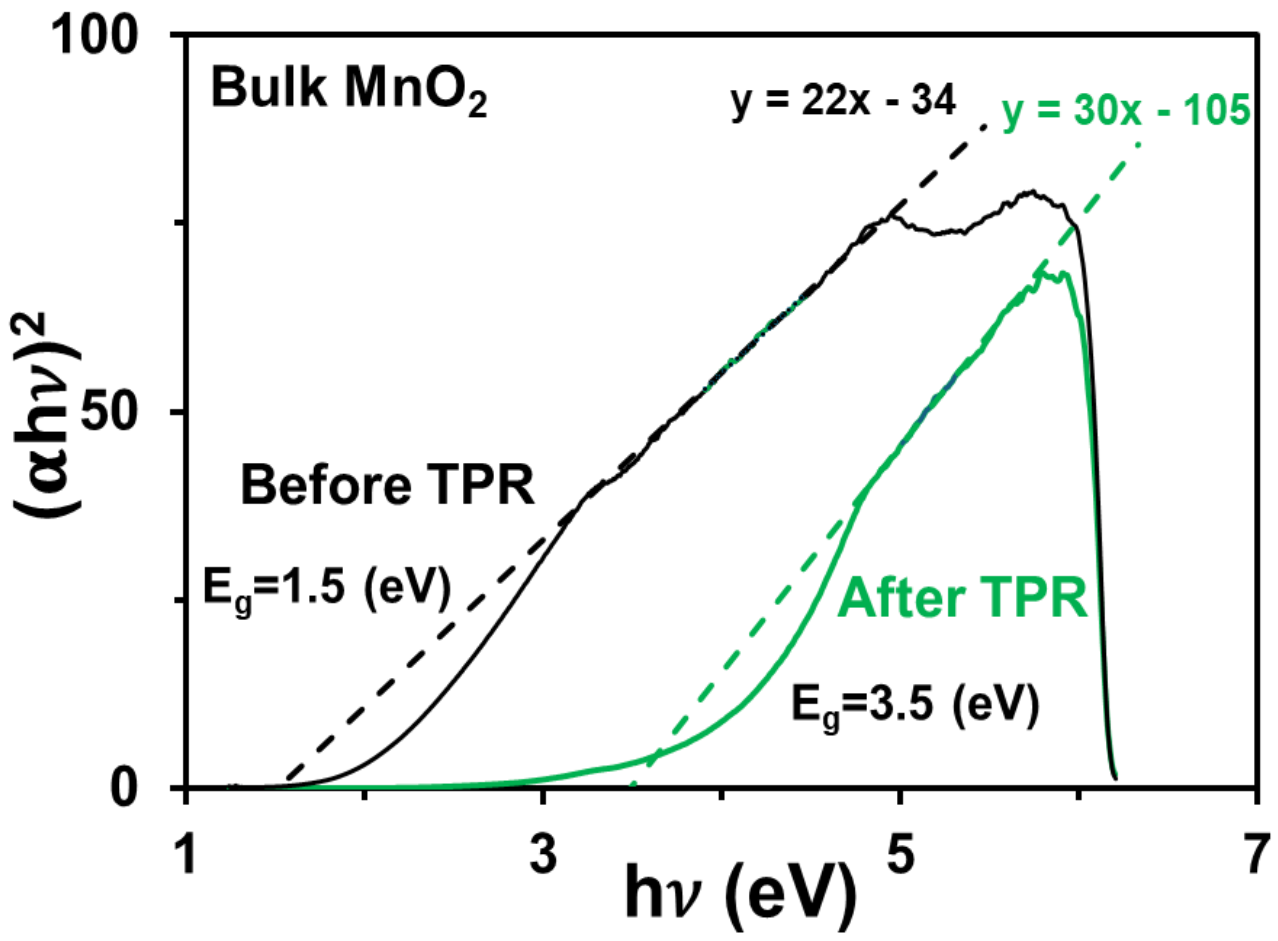

Figure S21: Tauc plot calculated from direct method Diffuse Reflectance UV-vis spectra of (A) freshly calcined bulk MnO_x (B) Reduced bulk MnO_x (MnO). Samples diluted with KBr and corresponding band gap (Eg) values calculated from the x-intercept of linear portion using the direct transition method.[20, 21] A KBr background collected immediately before the tests. Reduction was performed under 5% H_2/Ar at 400 °C.

4. References

(1) Yun, T. Y.; Battiste, A. M.; Pathickal Abraham, A.; Hart, K. D.; Chandler, B. D. Surface Entropy Mediated Hydrogen Spillover on Au/TiO2: Influences of Strongly Adsorbed Water on H2 Adsorption Thermodynamics. *Journal of the American Chemical Society* **2025**, *147* (33), 29908-29918. DOI: 10.1021/jacs.5c06813.

(2) Audrey M. Battiste [1] , T. Y. Y.; Kelle D. Hart [1] , R. J. S., Mohammad Hamidizirasefi [2] , Anela Pathickal Abraham [1] , Bert D. Chandler* [,1,2]. A quantitative examination of catalyst component impacts on hydrogen adsorption and spillover, Manuscript ID: ja-2026-089468.R3, 2026.

(3) Hart, K. D.; Hollobaugh, M. J.; Battiste, A. M.; Yun, T. Y.; Abraham, A. P.; Hamidizirasefi, M.; Loscher, I. M.; Chandler, B. D. Upside-Down Adsorption: The Counterintuitive Influences of Surface Entropy and Surface Hydroxyl Density on Hydrogen Spillover. *Journal of the American Chemical Society* **2024**, *146* (44), 30091-30103. DOI: 10.1021/jacs.4c07539.

(4) Mehrabadi, B. A. T.; Eskandari, S.; Khan, U.; White, R. D.; Regalbuto, J. R. Chapter One - A Review of Preparation Methods for Supported Metal Catalysts. In *Advances in Catalysis*, Song, C. Ed.; Vol. 61; Academic Press, 2017; pp 1-35.

(5) Di Castro, V.; Polzonetti, G. XPS study of MnO oxidation. *Journal of Electron Spectroscopy and Related Phenomena* **1989**, *48* (1), 117-123. DOI: https://doi.org/10.1016/0368-2048(89)80009-X.

(6) v4.0, O. i. l. u. C. C. A. L. *Atoms First 2e* OpenStax, Ed.; 2019.

(7) Cheraghi, A.; Hossein, Y.; and Safarian, J. Gaseous Reduction of Manganese Ores: A Review and Theoretical Insight. *Mineral Processing and Extractive Metallurgy Review* **2020**, *41* (3), 198-215. DOI: 10.1080/08827508.2019.1604523.

(8) Sarkar, A.; Schanche, T. L.; Wallin, M.; Safarian, J. Evaluating the Reaction Kinetics on the H2 Reduction of a Manganese Ore at Elevated Temperatures. *Journal of Sustainable Metallurgy* **2024**, *10* (4), 2085-2103. DOI: 10.1007/s40831-024-00964-6.

(9) Luther, G. W.; Thibault de Chanvalon, A.; Oldham, V. E.; Estes, E. R.; Tebo, B. M.; Madison, A. S. Reduction of Manganese Oxides: Thermodynamic, Kinetic and Mechanistic Considerations for One- Versus Two-Electron Transfer Steps. *Aquatic Geochemistry* **2018**, *24* (4), 257-277. DOI: 10.1007/s10498-018-9342-1.

(10) Seah, M. P. Summary of ISO/TC 201 Standard: VII ISO 15472 : 2001—surface chemical analysis—x-ray photoelectron spectrometers—calibration of energy scales. *Surface and Interface Analysis* **2001**, *31* (8), 721-723. DOI: https://doi.org/10.1002/sia.1076 (accessed 2025/06/05).

(11) Ilton, E. S.; Post, J. E.; Heaney, P. J.; Ling, F. T.; Kerisit, S. N. XPS determination of Mn oxidation states in Mn (hydr)oxides. *Applied Surface Science* **2016**, *366*, 475-485. DOI: https://doi.org/10.1016/j.apsusc.2015.12.159.

(12) Anderson, C.; Bard, A. J. Improved photocatalytic activity and characterization of mixed TiO2/SiO2 and TiO2/Al2O3 materials. *Journal of Physical Chemistry B* **1997**, *101* (14), 2611-2616, Article. DOI: 10.1021/jp9626982 Scopus.

(13) Aguado, J.; van Grieken, R.; López-Muñoz, M.-J.; Marugán, J. A comprehensive study of the synthesis, characterization and activity of TiO2 and mixed TiO2/SiO2 photocatalysts. *Applied Catalysis A: General* **2006**, *312*, 202-212. DOI: https://doi.org/10.1016/j.apcata.2006.07.003.

(14) López, R.; Gómez, R. Band-gap energy estimation from diffuse reflectance measurements on sol–gel and commercial TiO2: a comparative study. *Journal of Sol-Gel Science and Technology* **2012**, *61* (1), 1-7. DOI: 10.1007/s10971-011-2582-9.

(15) Mantilla, A.; Cipagauta Díaz, S.; Samaniego Benitez, E.; Tzompantzi Morales, F. J.; Navarrete Magaña, M. Design of MnOx/TiO2 Nanostructures for Photocatalytic Removal of 2,4-D Herbicide. *ChemistryOpen* **2025**, *14* (1), e202400154. DOI: https://doi.org/10.1002/open.202400154 (accessed 2025/06/05).

(16) Makuła, P.; Pacia, M.; Macyk, W. How To Correctly Determine the Band Gap Energy of Modified Semiconductor Photocatalysts Based on UV–Vis Spectra. *The Journal of Physical Chemistry Letters* **2018**, *9* (23), 6814-6817. DOI: 10.1021/acs.jpclett.8b02892.

(17) Zhang, T.; Li, B.; Wang, C.; Wu, S.; Zhu, S.; Jiang, H.; Zheng, Y.; Li, Z.; Cui, Z.; Zhang, Y.; et al. Tunning the bandgap of MnO2 homojunction by building active high-index facet to achieve rapid electron transfer for enhanced photocatalytic sterilization. *Journal of Materials Science & Technology* **2024**, *168*, 265-275. DOI: https://doi.org/10.1016/j.jmst.2023.05.059.

(18) Gangwar, D.; Rath, C. Structural, optical and magnetic properties of α- and β-MnO2 nanorods. *Applied Surface Science* **2021**, *557*, 149693. DOI: https://doi.org/10.1016/j.apsusc.2021.149693.

(19) Cipriano, L. A.; Di Liberto, G.; Tosoni, S.; Pacchioni, G. Band Gap in Magnetic Insulators from a Charge Transition Level Approach. *Journal of Chemical Theory and Computation* **2020**, *16* (6), 3786-3798. DOI: 10.1021/acs.jctc.0c00134.

(20) Jha, A.; Thapa, R.; Chattopadhyay, K. K. Structural transformation from Mn3O4 nanorods to nanoparticles and band gap tuning via Zn doping. *Materials Research Bulletin* **2012**, *47* (3), 813-819. DOI: https://doi.org/10.1016/j.materresbull.2011.11.057.

(21) Hosny, N. M.; Dahshan, A. Facile synthesis and optical band gap calculation of Mn3O4 nanoparticles. *Materials Chemistry and Physics* **2012**, *137* (2), 637-643. DOI: https://doi.org/10.1016/j.matchemphys.2012.09.068.
